# Molecular intrinsic subtypes, genomic, and immune landscapes of *BRCA*-proficient but HRD-high ER-positive/HER2-negative early breast cancers

**DOI:** 10.1186/s13058-022-01572-6

**Published:** 2022-11-18

**Authors:** Elise Ballot, Loïck Galland, Hugo Mananet, Romain Boidot, Laurent Arnould, Isabelle Desmoulins, Didier Mayeur, Courèche Kaderbhai, Silvia Ilie, Audrey Hennequin, Anthony Bergeron, Valentin Derangère, François Ghiringhelli, Caroline Truntzer, Sylvain Ladoire

**Affiliations:** 1 Department of Medical Oncology, Georges-François Leclerc Center - UNICANCER, 1 Rue du Professeur Marion, 21000 Dijon, France; 2 Platform of Transfer in Biological Oncology, Georges-François Leclerc Cancer Center, Dijon, France; 3 University of Burgundy-Franche Comté, Besançon, France; 4 Bioinformatic Core Facility Georges, François Leclerc Cancer Center, Dijon, France; 5 Department of Pathology and Tumor Biology, Georges François Leclerc Center, Dijon, France; 6 Centre de Recherche INSERM LNC-UMR1231, Dijon, France; 7 Genomic and Immunotherapy Medical Institute, Dijon University Hospital, Dijon, France

**Keywords:** Early breast cancer, ER-positive breast cancer, *BRCA*, Exome, NGS, HRD score, Signature 3, Homologous recombination

## Abstract

**Purpose:**

The vast majority of research studies that have described the links between DNA damage repair or homologous recombination deficiency (HRD) score, and tumor biology, have concerned either triple negative breast cancers or cancers with mutation of *BRCA 1/2.* We hypothesized that ER + /HER2- early breast tumors without *BRCA 1/2* mutation could have high HRD score and aimed to describe their genomic, transcriptomic, and immune landscapes.

**Patients and methods:**

In this study, we reported *BRCA 1/2* mutational status, HRD score, and mutational signature 3 (S3) expression, in all early breast cancer (eBC) subtypes from the TCGA database, with a particular focus in ER + /HER2-. In this subtype, bioinformatics analyses of tumor transcriptomic, immune profile, and mutational landscape were performed, according to HRD status. Overall survival (OS), progression free-interval (PFI), and variables associated with outcome were also evaluated.

**Results:**

Among the 928 tumor samples analyzed, 46 harbored *BRCA 1/2* mutations, and 606 were ER + /HER2- (of which 24 were *BRCA 1/2* mutated). We found a subset of *BRCA*-proficient ER + /HER2— eBC, with high HRD score. These tumors displayed significantly different immune, mutational, and tumor molecular signatures landscapes, compared to *BRCA*-mutated and *BRCA*-proficient HRD-low tumors. Outcome did not significantly differ between these 3 groups, but biological factors associated with survival are not the same across the 3 entities.

**Conclusion:**

This study highlights possible novel biological differences among ER + /HER2- breast cancer related to HRD status. Our results could have important implications for translational research and/or the design of future clinical trials, but require prospective clinical evaluation.

**Supplementary Information:**

The online version contains supplementary material available at 10.1186/s13058-022-01572-6.

## Introduction

Breast cancer is a very heterogeneous disease, defined by different entities according to histological, genomic, and transcriptomic classifications. Mutations in *BRCA 1* and *BRCA 2* are the main cause of inherited breast cancer, but may also be involved in the oncogenesis of sporadic forms, via purely somatic mutations. The proteins encoded by these genes play an essential role in maintaining DNA integrity, helping double-strand DNA break repair during the process of homologous recombination (HR) [1]. A deficiency in HR within tumor cells can occur due to a germline or somatic mutation of *BRCA 1/2*, but also via multiple other genomic or epigenetic alterations involving the other actors in this cellular process, thus resulting in homologous recombination deficiency (HRD) in sporadic tumors.

From a therapeutic point of view, a deficit in the HR process also constitutes the “Achilles heel” of the tumor cell. It is important to know this, as it makes the tumor more sensitive to agents generating double-stranded DNA lesions, such as platinum-based chemotherapies and poly (ADPribose) polymerase (PARP) inhibitors (PARPi) [1]. Several PARPi have been developed, initially for the treatment of ovarian cancer [2–4], while more recently, agents such as olaparib and talazoparib have been also approved for the treatment of metastatic breast cancer with *BRCA1/2* germline mutation (g*BRCA* 1/2) [5, 6].

Furthermore, these HR-deficient tumors are considered to be more immunogenic than HR proficient tumors [7–9]. Indeed, tumors with *BRCA 1/2* mutations are more genomically unstable and therefore potentially have a higher number of non-synonymous mutations, and thus, tumor neoantigens [8, 9]. Moreover, these tumors are characterized by higher expression of PD-L1, inflammatory signatures of an immune response, and greater infiltration by lymphocytes [7, 9]. It is possible that part of the therapeutic effect of PARPi is mediated by activation of the immune response via the STING pathway [10]. There is therefore a strong rationale for combining PARPi and immunotherapy for these tumors with *BRCA 1/2* mutations.

In breast cancer, 15% of triple negative cancers (TNBC) have a deficiency in HR attributable to a germline *BRCA 1/2* mutation [11], but 40% of all TNBCs are characterized by HRD in the absence of germline *BRCA 1/2* mutation [12, 13]. A major clinical challenge is therefore to identify patients without *BRCA 1/2* mutation, but whose tumor has HRD (“*BRCA*ness” phenotype), and who might therefore benefit from the same innovative therapeutic approaches [14].

In addition to the search for point mutations within the genes involved in HR thanks to dedicated panels, the analysis of mutational signatures associated with HRD such as “signature 3” (S3) described by Alexandrov et al. [15], or signatures designed to capture “genomic scars” associated with HRD, is commonly used to identify tumors that may benefit from platinum or PARPi treatment [16]. Among the commonly used genomic scar signatures, the “myChoice HRD” test (which is a combination of LOH, LST, and TAI scores) defines HRD-positive tumors based on a HRD score ≥ 42[17].

For breast cancers, an important question is the extent to which a high HRD score is associated with the same sensitivity to DNA-damaging agents and immunologic characteristics as *BRCA 1/2*-mutated tumors. To date, the vast majority of studies that have described the links between DNA damage repair or HRD score, sensitivity to platinum or PARPi, and tumor immunogenicity have concerned either triple negative cancers, or cancers with mutation of *BRCA 1* or *BRCA 2.*

In this work, we investigated ER + /HER2- tumors, a population of breast cancers less frequently treated by PARPi, platinum-based chemotherapy, and/or associations with immunotherapy. As we recently described a subgroup of metastatic luminal BC that were *BRCA-*proficient but with a high HRD score[18], we sought to describe the genomic, transcriptomic, and immunological landscapes of these tumors at the early stage in greater detail, in relation to the *BRCA 1/2* mutation and HRD status.

## Results

### Homologous recombination biomarker tumor status in Breast Cancer TCGA cohort

Among the 928 tumor samples analyzed, 46 patients (5%) carried *BRCA 1/2* mutations (32 with germline mutations and 14 with somatic mutations). Mean HRD score in the whole cohort was 25.80 $\pm$ 19.87. Using the classical cutoff value of 42 [16], high HRD score was found in 133/882 (15%) *BRCA 1/2 WT* patients and in 31/46 (67%) *BRCA 1/2*-mutated tumors (24 with germline mutations and 7 with somatic mutations) (Additional file 1: supplemental table 1).

**Fig. 1:**
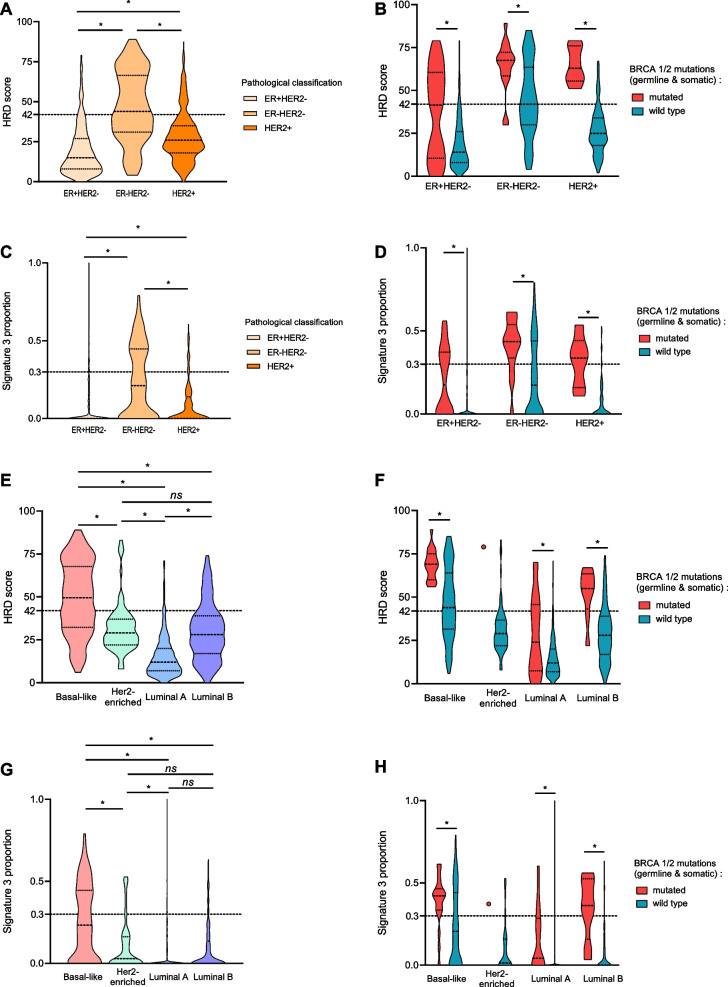
Distributions of HRD score and S3 proportion according to *BRCA* 1/2 mutational status and breast cancer subtypes (standard pathological classification or PAM50 subtypes) considering the whole cohort (*n* = 928). **A-C** Violin plots representing the distribution of HRD score (**A**) and signature 3 proportion (**C**) according to breast cancer standard pathological classification. **B-D** Violin plots representing the distribution of HRD score (**B**) and signature 3 proportion (**D**) according to breast cancer standard pathological classification and *BRCA* 1/2 mutational status. *Wilcoxon p-value < 0.05. **E–G** Violin plots representing the distribution of HRD score (**E**) and signature 3 proportion (**G**) according to PAM50 subtypes. *Wilcoxon p-value < 0.05. **F–H** Violin plots representing the distribution of HRD (**F**) and signature 3 proportion (**H**) score according to PAM50 subtypes and *BRCA* 1/2 mutational status

We first considered pathological subtypes, namely estrogen receptor-positive/HER2 negative (ER + /HER2-; *n* = 606 patients, 66%), HER2 + (*n* = 157 patients, 17%) and triple negative (*n* = 165 patients, 17%) early breast cancer (eBC). We compared HRD score and S3 expression according to these pathological subtypes (Fig. 1A and 1C, respectively). A significant association was found between the median HRD score and *BRCA 1/2* mutational status (median HRD score, respectively, 57.2 for patients with *BRCA 1/2* mutation and 20 for patients with *WT* status, *p *value < 0.001) (Additional file 2: supplemental figure 1A (S1A)). The same observation was made in each BC pathological subtype (Fig. 1B). Using the classical cutoff value of 0.3 [19], a high level of S3 was found in 100/882 (11.3%) *BRCA 1/2 WT* tumors and in 27/46 (58.7%) *BRCA 1/2*-mutated tumors (22 with germline mutations and 5 with somatic mutations). A significant association was found between median expression of mutational S3 and *BRCA 1/2* mutational status (median S3 score, respectively, 0.35 for patients with *BRCA 1/2* mutation and 0 for patients with *WT* status, *p *value < 0.001) (Additional file 2: supplemental figure 1C (S1C)). Higher expression of mutational S3 was found in *BRCA* 1/2-mutated tumors, in ER + /HER2- (*p* < 0.001), HER2 + (*p* < 0.001) and in triple negative subtypes (*p* = 0.002) (Fig. 1D).

Next, we considered intrinsic molecular subtypes (according to PAM50), namely basal-like (164 patients, 18%), HER2-enriched (77 patients, 8%), luminal A (492 patients, 53%) or luminal B (195 patients, 21%) tumors. Of note, tumors normal-like subtypes (*n* = 36) were excluded from all the analyses. We aimed to compare HRD score and S3 expression according to intrinsic molecular subtypes (PAM50), and ER expression among the different intrinsic subtypes. The highest levels of HRD score and S3 were found in the basal-like PAM50 subtype, while the lowest level were found in the Luminal A subtype (Fig. 1E and 1G). No significant difference was found in HRD scores between ER + and ER− tumors in the majority of PAM50 subtypes, except for the HER2 subtype (respectively, mean HRD score of 28 for patients with ER + status and 34 for patients with ER*-* status, *p *value = 0.03) (Additional file 2: supplemental figure 1B (S1B)). There was no difference in S3 levels between ER + and ER- tumors among the different PAM50 intrinsic subtypes (Additional file 2: supplemental figure 1D (S1D)). Higher levels of HRD score and S3 were found in *BRCA* 1/2-mutated tumors in each PAM50 intrinsic subtype (Fig. 1F and 1H).

In order to differentiate between BRCA1/2 germline or somatic mutations, same analyses were performed concerning the distribution of the HRD score and S3 proportion according to *BRCA* 1/2 mutational status (germline or somatic) considering the whole cohort (*n* = 928) (Additional file 3: supplemental figure 2E–F (S2 E–F)) or by breast cancer subtypes (standard pathological classification or PAM50 subtypes) (Additional file 3: supplemental figure 2A–D (S2 A–D)).

**Fig. 2:**
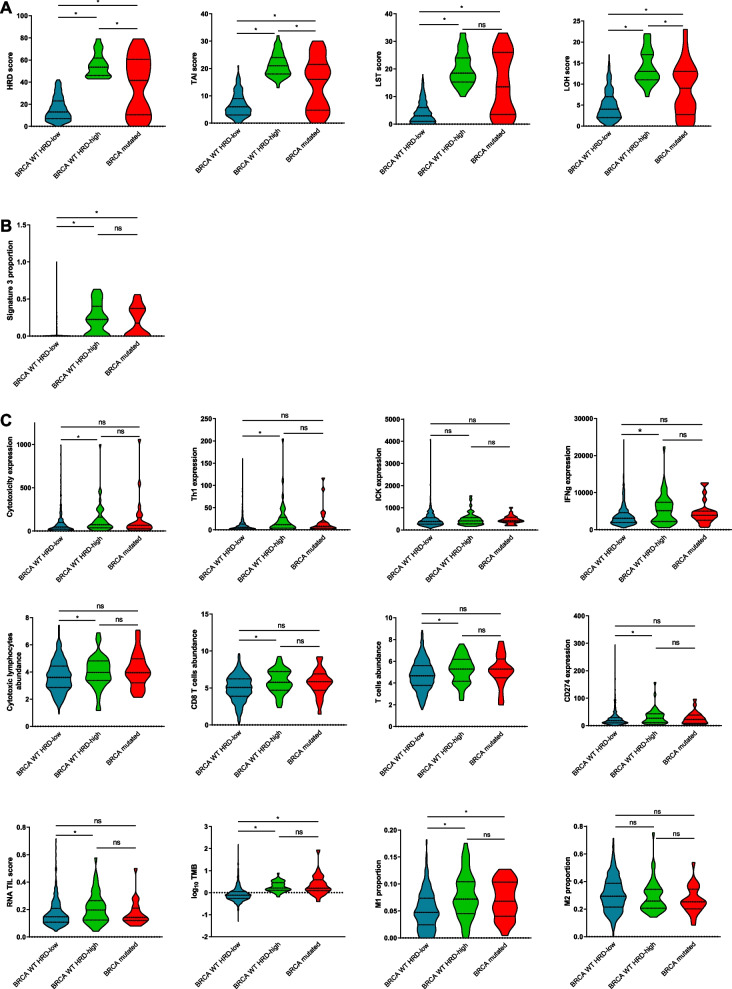
Association of genomic features quantifying tumor HRD and immunological characterization in ER + /HER2- tumors (*n* = 606). Violin plots representing the distribution of HRD, TAI, LST and LOH scores (**A**), signature 3 proportion (**B**), and immune signatures (**C**) according to HRD level and *BRCA* 1/2 mutational status in ER + /HER2- tumors. *Wilcoxon p-value < 0.05

Finally, we considered exclusively ER + /HER2- tumors (pathological subtype), distributed according to PAM50 subtypes as follows: basal-like (17 patients, 3%, including 1 *BRCA 1/2*-mutated tumor), HER2-enriched (5 patients, 1%, including 1 *BRCA 1/2*-mutated tumor), luminal A (426 patients, 70%, including 16 *BRCA 1/2*-mutated tumors) or luminal B (158 patients, 26%, including 7 *BRCA 1/2*-mutated tumors): higher levels of HRD score and S3 were only found in luminal B *BRCA* 1/2-mutated tumors compared to WT tumors (Additional file 4: supplemental figure 3A–B (S3 A–B), Additional file 1: supplemental table 1).

**Fig. 3:**
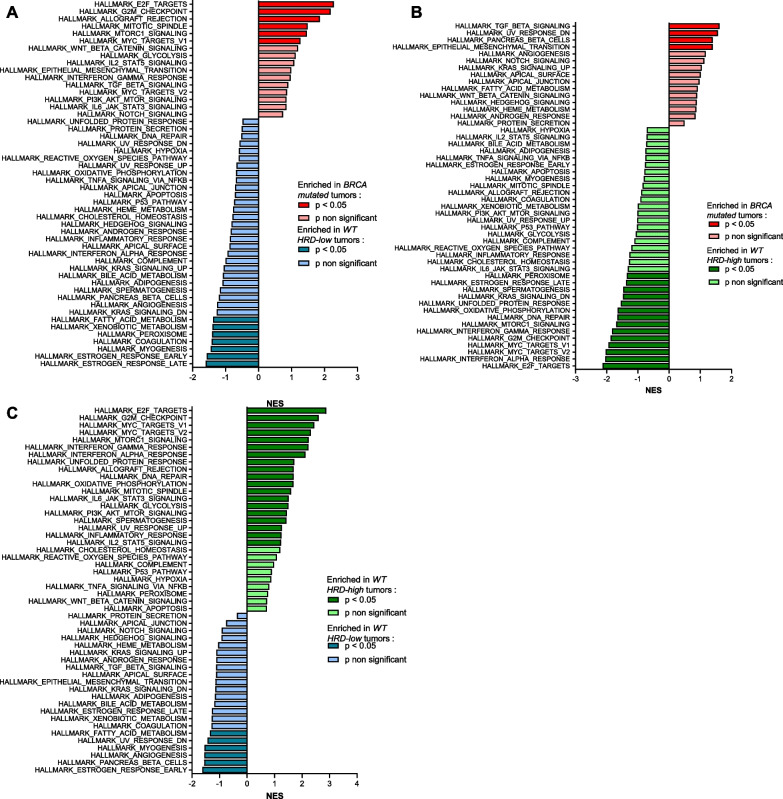
Gene set enrichment analysis in ER + /HER2- tumors (*n* = 606). **A-C** Gene set enrichment analysis (GSEA) barplots for HALLMARK collection. Bars represent the normalized enrichment score (NES), using genes differentially expressed between: (**A**) patients with *BRCA*-mutated tumors vs patients with *BRCA* WT HRD-low tumors, (**B**) patients with *BRCA*-mutated tumors vs patients with *BRCA* WT HRD-high tumors, and (**C**) patients with *BRCA* WT HRD-low tumors vs patients with *BRCA* WT HRD-high tumors. Red bars: patients with *BRCA*-mutated tumors, green bars: patients with *BRCA* WT HRD-high tumors, blue bars: patients with *BRCA* WT HRD-low tumors. Light colors represent non-significant p values and dark colors significant p values (< 0.05)

Taken together, these results make it possible to describe the HRD and S3 level in the different subtypes (pathological or molecular intrinsic) of breast cancer, according to the expression of hormone receptors, and *BRCA* deficient or proficient status. The distribution of HRD score, PAM50 intrinsic subtypes, *BRCA 1/2* mutations, Signature 3 proportion, and ER status in whole cohort is summarized in Additional file 5: supplemental figure 4A (S4A). As expected, HRD-high tumors essentially comprise tumors of the basal-like subtype, the majority (but not all) of tumors with BRCA mutation, and with a high 3 signature.

**Fig. 4:**
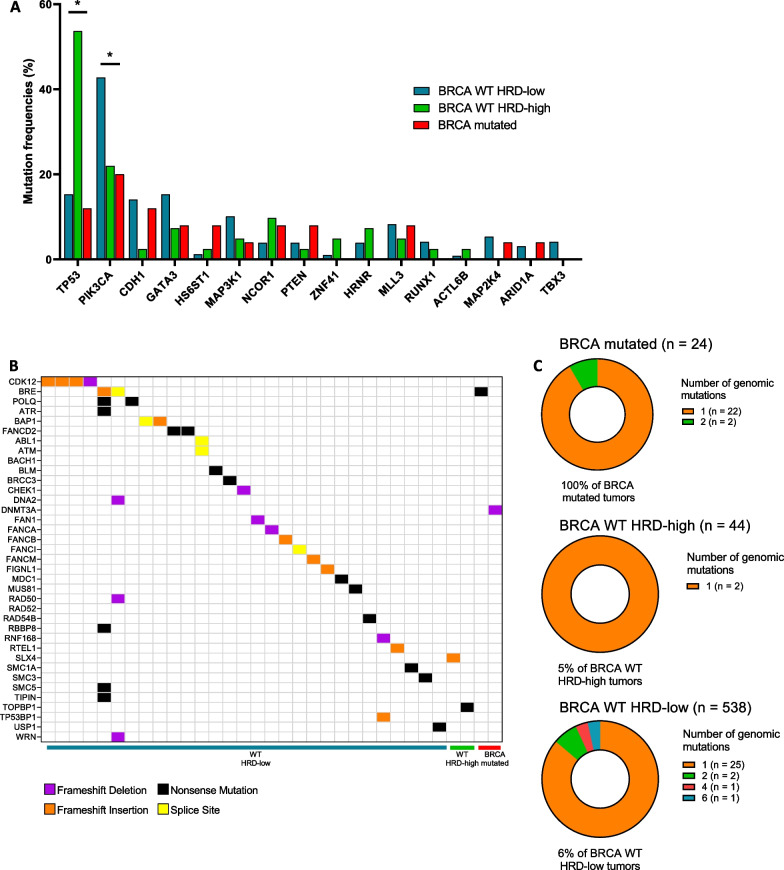
Mutational landscape in ER + /HER2- tumors (*n* = 606). **A** Barplots representing the frequencies of most frequent mutated driver genes, according to HRD and *BRCA* 1/2 mutational status. * Fisher's exact test p value < 0.05. **B** Landscape of pathogenic or likely pathogenic somatic mutations of genes involved in the homologous recombination pathway, *BRCA*1/2 excluded. **C** Pie charts of number of pathogenic or likely pathogenic somatic mutations of genes involved in the homologous recombination pathway according to HRD and *BRCA* 1/2 mutational status

### A subset of BRCA-proficient ER + /HER2- breast cancers harbored high HRD score

In metastatic ER + /HER2- BC, we recently described a subgroup of *BRCA*-proficient tumors, but with high HRD score [18]. Therefore, we investigated whether such a BC subtype also existed in early stage BC. Considering exclusively ER + /HER2- tumors (*n* = 606) from the TCGA, we found 24 tumors with *BRCA 1/2* mutation (4%), and also a group with high HRD score (> 42), but without *BRCA 1/2* mutation (44 tumors, 7.3%). This enabled us to study these 3 distinct groups of ER + /HER2- tumors for the remainder of the experiments, i.e., *BRCA* mutated, *BRCA*-proficient (WT) HRD high, and *BRCA*-proficient (WT) HRD low. Considering molecular intrinsic subtypes according to the PAM50 classification, these 3 subgroups showed a significantly different distribution of molecular intrinsic subtypes (Additional file 5: supplemental figure 4B–E (S4 B–E)): *BRCA*-mutated tumors were mainly luminal A (62.5%) and luminal B (29%). *BRCA* WT HRD-low tumors were mainly luminal A (74%), and basal-like in only 2% of case. Conversely, in *BRCA* WT HRD-high tumors, we found 16% of basal-like, 54.5% of luminal B but only 29.5% of luminal A tumor intrinsic subtypes. Accordingly, we found a significantly positive correlation between HRD score and expression of a proliferation signature [20] (Additional file 6: supplemental figure 5A (S5A)). Tumors with high HRD score have higher proliferation signature (Additional file 6: supplemental figure 5B (S5B)), and these differences were seen in each molecular subtype (PAM50) (Additional file 6: supplemental figure 5C (S5C)).

**Fig. 5:**
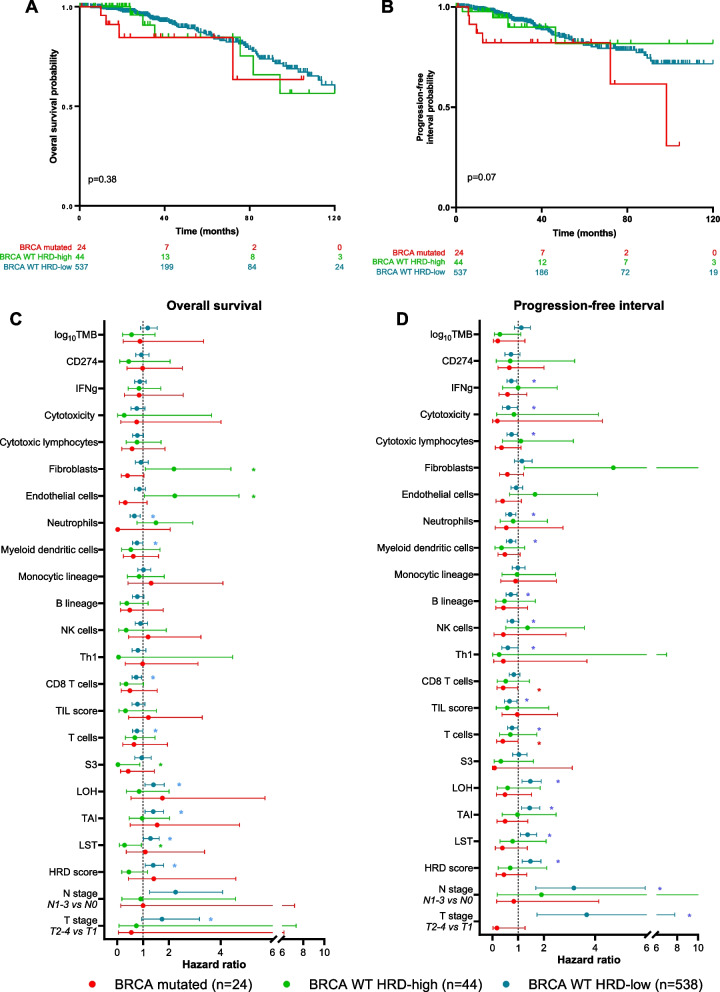
Progression-free interval and overall survival according to *BRCA 1/2*-mutated status and HRD score level in ER + /HER2- tumors (*n* = 606). **A**, **B** Kaplan–Meier curves of overall survival (**A**) and progression-free interval (**B**) according to *BRCA 1/2* mutational status and HRD score level. Red curves: patients with *BRCA*-mutated tumors, green curves: patients with *BRCA WT* HRD-high tumors, blue curves: patients with *BRCA WT* HRD-low tumors. Ticks denote censored data. **C, D** Forest plots of hazard ratio (HR) for the association of the clinical variables and immune scores with overall survival (**C**) and progression-free interval (**D**) according to *BRCA 1/2* mutational status and HRD score level. Red lines: patients with *BRCA*-mutated tumors, green lines: patients with *BRCA WT* HRD-high tumors, blue lines: patients with *BRCA WT* HRD-low tumors. Horizontal lines represent 95% CI. Each point represents estimated HR. The dashed vertical line indicates HR = 1. *Wald-test p-value < 0.05

In *BRCA*-proficient (WT) HRD-high tumors, HRD scores were even higher than those in *BRCA*-mutated tumors (Fig. 2A and Additional file 7: supplemental figure 6A (S6A)), driven by higher TAI and LOH scores. These *BRCA* WT HRD-high tumors had S3 levels comparable to *BRCA*-mutated tumors, and higher than *BRCA* WT HRD-low tumors (Fig. 2B and Additional file 7: supplemental fig 6B (S6B)). We next compared *BRCA 1/2* expression between patients with *BRCA*-mutated tumors, *BRCA* WT HRD-high and *BRCA* WT HRD-low tumors. We did not find a significant difference in *BRCA2* expression between *WT* HRD-high and *WT* HRD-low tumors, whereas *BRCA2* expression was logically significantly lower in *BRCA*-mutated tumors (Additional file 8: supplemental figure 7 (S7)). Thus, the difference in HRD score between these 2 groups does not seem to be explained by epigenetic regulation of *BRCA2* gene. Same analyses were performed within the different molecular subtypes to investigate the expression of *BRCA1* and *BRCA2* among the *BRCA*-mutated, WT HRD-high, and WT HRD-low groups (Additional file 9: supplemental figure 8 (S8)). Here again, no differences of *BRCA1* or *BRCA2* expression were seen between BRCA WT HRD-high or HRD-low tumors, whatever PAM50 subtype.

### Immunological tumor landscape of ER + /HER2- breast cancers according to BRCA and HRD status

Since different immune microenvironments have been described between *BRCA* WT and *BRCA*-mutated BC [21], we next evaluated the immunological landscape of our 3 different groups of eBC even within ER + /HER2- tumors (Fig. 2C and Additional file 7: supplemental figure 6C (S6C)). No significant difference was found in any of our analyses between *BRCA 1/2*-mutated and *WT* HRD-high tumors. These results indicate that this small subset of HRD high *BRCA 1/2* proficient ER + /HER2- eBC actually had immunological features comparable to those of their *BRCA 1/2*-mutated counterparts. On the contrary, several significant differences in tumor immune profile were found between WT HRD-high and WT HRD-low tumors, notably T cell abundance, CD8 T cell abundance, cytotoxic T cell abundance, and cytotoxicity expression signature, but also TIL abundance (evaluated by RNA TILS score signature), Th1 lymphocyte gene expression signature, interferon-γ expression signature, were all significantly higher in *BRCA WT* HRD-high compared to WT HRD-low tumors, suggesting a different immunological cellular landscape between these two groups, with WT HRD-high tumors having higher tumor immune infiltrate of cytotoxic lymphocytes. Moreover, these WT HRD-high tumors also appeared to have significantly higher tumor mutational burden (TMB), and higher *CD274* (PD-L1) expression than WT HRD-low tumors. Concerning macrophages subpopulations, M1 were found to be higher in *BRCA*-mutated tumors and *BRCA* WT HRD-high when compared to *BRCA* WT HRD-low cases (with no difference regarding M2 subpopulation). Of note, no significant difference was observed in terms of parameters associated with B or NK cell lineage (not shown), or in terms of other (than PD-L1) inhibitory immune checkpoint expression. No difference was noted among BRCA-mutated tumors, when HRD-high and HRD-low cases were compared (Additional file 7: supplemental figure 6 (S6)), but the number of BRCA-mutated tumors with low HRD score was very limited.

Collectively, these results therefore show that among ER + / HER2- tumors, *BRCA*-proficient HRD-high tumors have an immunological landscape comparable to that of *BRCA*-mutated tumors, and apparently more favorable to therapeutic approaches using immune checkpoint blockers, compared to *BRCA* WT HRD-low tumors.

### Tumor cellular pathways of ER + /HER2- breast cancers according to BRCA and HRD status

To explore the underlying mechanisms of our findings, we next aimed to determine whether different cellular/oncogenic pathways could be associated, and differentially expressed in these 3 different subtypes of ER + /HER2- tumors. Pathway enrichment analysis using the Hallmark gene sets from MSigDB revealed strong differences between the 3 different HRD-status eBC (Fig. 3A–C). Briefly, compared to *BRCA* WT HRD-low BC, *BRCA*-mutated tumors were enriched in proliferation module gene sets (*E2F target, G2M checkpoint, MYC targets, mitotic spindle signatures*), but also in immune pathway (*allograft_rejection signature*), and *mTORC1 pathway* (Fig. 3A). Conversely, *BRCA* WT HRD-low were significantly more enriched in molecular signature modules related to estrogen response (*early and late response*).

Interestingly, *BRCA* WT HRD-high tumors also differed from *BRCA*-mutated tumors, but via different biological cellular processes (Fig. 3B): For example, *BRCA* WT HRD-high tumors appeared to be enriched in immune modules (*allograft_rejection signature, interferon gamma, and interferon alpha response*), proliferation module genes (*E2F target, G2M checkpoint, MYC targets, mitotic spindle signatures*), but also *DNA repair* (thereby confirming our previous results obtained for HRD scores), and other potential therapeutic targets like *estrogen response* or *MTORC1*, or *KRAS signaling*.

Finally, the main differences in molecular pathway signatures between *BRCA* WT tumors (HRD high vs HRD low) were statistically significant enrichment in numbers of immune pathways in WT HRD-high tumors (*allograft_rejection signature, interferon gamma, and interferon alpha response, IL6_JAK_STAT3, inflammatory response, IL2_STAT5 signaling*) thereby confirming our previous results concerning the tumor immune landscape, but also *MTORC1*, and logically *DNA repair signaling* in WT HRD-high tumors, compared with their WT HRD-low counterparts (which remained enriched in some classical pathways involved in luminal tumor biology, like *estrogen response*) (Fig. 3C).

Collectively, these results show that in addition to differences in immunological tumor profile, there seems to be a wider range of biological processes that differ between these 3 subtypes of ER + /HER2- BC. These results also provide a biological rationale for selecting appropriate pharmacological targeting in each of these different subtypes.

### Tumor mutational landscape of ER + /HER2- breast cancers according to BRCA and HRD status

We then aimed to determine the tumor mutational landscape in these 3 groups of ER + /HER2- tumors. Only driver genes were considered in this analysis. The most frequent mutated driver genes in each group are presented in Fig. 4A. Patients with *WT* HRD-high tumors have a different mutational landscape to that of *BRCA 1/2*-mutated tumors or *WT* HRD-low tumors. We found that *BRCA* WT HRD-high tumors presented a higher rate of *TP53* mutations compared to *BRCA* WT HRD-low tumors or *BRCA*-mutated tumors. Conversely, *PIK3CA, GATA3, MAP3K1, CDH1* mutations appeared to be more frequent in *BRCA* WT HRD-low tumors compared to *BRCA* WT HRD-high tumors.

Considering exclusively genes involved in homologous recombination (HR) (except *BRCA1* and *BRCA2*), we found that 33 (6%) of the 541 samples with mutations available presented at least one somatic gene alteration (Fig. 4B). However, no significant difference was found in the number of somatic mutations in HR-associated genes between the *BRCA 1/2*-mutated, WT HRD-high, and WT HRD-low groups (Additional file 10: supplemental figure 9 (S9)).

A complete description of the mutations found in each group is presented in Fig. 4B.

Only 2 *BRCA*-mutated tumors had a second HR-gene mutation (Fig. 4C, upper panel). Similarly, in *BRCA* WT HRD-high tumors, we found only 2 cases (5% of these tumors) harboring a single HR-gene mutation (Fig. 4C, middle panel). In *BRCA WT* HRD-low tumors, we found the same proportion (*n* = 29; 6%) of cases with at least one HR-gene mutation (Fig. 4C, lower panel). In the vast majority of cases, it was a single mutation (*n* = 25), but we also found 4 tumors with ≥ 2 HR-gene mutations.

### Outcome of ER + /HER2- early breast cancers according to BRCA and HRD status

We finally aimed to determine whether these 3 groups of ER + /HER2- tumors, stratified according to HRD status, would have different prognosis, and how this new biological segmentation could account for the risk of relapse and death, compared to previously described, classical prognostic factors, and biological factors related to the tumor microenvironment.

We first sought to define the biological variables associated with the progression-free interval (PFI) in the ER + /HER2- tumors cohort (Table 1).

**Table 1:** Factors associated with progression-free interval by univariate and multivariate analysis using Cox models with lasso penalty

Variables	Threshold	Univariate		Multivariate	
		HR [95 % CI]	*p *value	Adjusted *p *value	HR [95 % CI]
Group					
*BRCA WT HRD-low*	-	1			
*BRCA WT HRD-high*	-	0.76 [0.27;2.1]	0.59	0.61	0.83 [0.78; 0.87]
*BRCA mutated*	-	2.56 [1.1;5.93]	0.03	0.05	1.76 [1.72; 1.80]
HRD score	42.00	1.06 [0.48;2.32]	0.89	0.89	
TAI score	9.00	1.94 [1.21;3.11]	0.01	0.02	1.38 [1.34; 1.42]
LST score	4.00	1.95 [1.21;3.13]	0.01	0.02	1.19 [1.18; 1.20]
LOH score	3.00	1.33 [0.81;2.17]	0.26	0.32	
Signature 3 proportion	0.30	0.67 [0.27;1.66]	0.38	0.43	
Cytotoxicity	59.10	0.49 [0.29;0.81]	0.01	0.02	
Th1	7.96	0.5 [0.29;0.86]	0.01	0.03	
CTL	265.94	0.44 [0.25;0.76]	0.00	0.02	
ICK	430.60	0.34 [0.19;0.61]	0.00	0.00	0.48 [0.47; 0.50]
IFNg	2894.00	0.55 [0.34;0.89]	0.01	0.03	0.98 [0.97; 1.00]
CD274 (PD-L1)	17.04	0.69 [0.43;1.1]	0.12	0.18	
log10 TMB	− 0.12	1.35 [0.83;2.2]	0.22	0.29	
T cells	4.07	0.51 [0.32;0.81]	0.01	0.02	0.89 [0.88; 0.90]
CD8 T cells	5.09	0.49 [0.3;0.79]	0.00	0.02	
Cytotoxic lymphocytes	3.73	0.39 [0.23;0.66]	0.00	0.00	0.65 [0.65; 0.65]
NK cells	1.61	0.56 [0.33;0.95]	0.03	0.06	
B lineage	3.58	0.52 [0.32;0.84]	0.01	0.02	
Monocytic lineage	7.64	0.83 [0.52;1.33]	0.44	0.47	
Myeloid dendritic cells	3.89	0.45 [0.28;0.72]	0.00	0.01	0.75 [0.74; 0.76]
Neutrophils	4.94	0.47 [0.29;0.76]	0.00	0.02	0.67 [0.66; 0.69]
Endothelial cells	7.32	0.78 [0.49;1.26]	0.31	0.38	
Fibroblasts	13.20	1.64 [1.02;2.63]	0.04	0.07	2.35 [2.25; 2.45]
TILS	0.15	0.54 [0.32;0.9]	0.02	0.04	

HR: Hazard ratio (high vs low level for dichotomized variables), CI: confidence interval, adj *p *value : adjusted *p *value, LOH: loss of heterozygosity, TAI: telomeric allelic imbalance, LST: large scale state transition, CTL: cytotoxic T cell lymphocytes, ICK: inhibitory immune checkpoint, IFNg: interferon gamma, TMB: tumor mutational burden, NK: natural killer, TILS: tumor infiltrating lymphocyte signature

By univariate analysis, patients with *BRCA*-mutated tumors had poorer PFI compared to patients with *BRCA* WT HRD-low tumors (HR: 2.56 [1.1; 5.93], p = 0.05). No significant difference was found between *BRCA* WT HRD-low and *BRCA* 1/2 WT HRD-high tumors (HR: 0.76 [0.27; 2.1], p = 0.61). (By the same, the direct comparison between BRCA-mutated and BRCA WT HRD-high cases found no significant difference in terms of PFI (not shown).) Two HRD-related variables were associated with shorter PFI, namely TAI (HR = 1.94 [1.21; 3.11], p = 0.02) and LST (HR = 1.95 [1.21; 3.13], p = 0.02). 13 biological variables, mainly immune variables (interferon gamma, T cell receptor (TCR), cytotoxicity (CYTOX), TH1, cytotoxic T cell lymphocytes (CTL), and inhibitory immune checkpoint (ICK)) signatures, T cells, CD8 T cells, cytotoxic lymphocytes, natural killer cells, B lineage, myeloid dendritic, and neutrophils abundances and TILS score, were associated with better PFI. Conversely, fibroblast abundance (HR = 1.64 [1.02; 2.63], p = 0.07) tended to be associated with shorter PFI.

By multivariate analysis, using a LASSO model, we found that TAI score (HR = 1.38 [1.34; 1.42]), LST score (HR = 1.19 [1.18; 1.20]), and fibroblast abundance (HR = 2.35 [2.25; 2.45]) were associated with shorter PFI. Inhibitory immune checkpoint (ICK) signature (HR = 0.48 [0.47; 0.50]), T cells (HR = 0.89 [0.88; 0.90]), cytotoxic lymphocytes (HR = 0.65 [0.65; 0.65]), myeloid dendritic cells (HR = 0.75 [0.74; 0.76]), IFNg (HR = 0.98 [0.97; 1.00]), and neutrophil (HR = 0.67 [0.66; 0.69]) abundance were associated with better PFI. Same trends in these exploratory analyses were observed when overall survival (OS) was studied (Additional file 11: supplemental table 2).

Concerning survival in our 3 groups of patients, median overall survival (OS) was not reached for patients with *BRCA 1/2*-mutated tumors or for patients with WT HRD-high tumors and was 130 months for patients with WT HRD-low tumors (Fig. 5A). Regarding PFI, the median PFI was 98 months for patients with *BRCA 1/2*-mutated tumors, not reached for patients with WT HRD-high tumors, and 168 months for patients with WT HRD-low tumors (Fig. 5B). There was no statistically significant difference in terms of OS or PFI between the 3 groups of tumors.

We then investigated in an exploratory analysis, whether OS and PFI were associated with different prognostic factors within these three tumor groups (Fig. 5C-D). Neither HRD score nor any its 3 components (LST, TAI, and LOH) were associated with different outcome in any of the 3 groups of tumors. The same was true for S3 level, except for *BRCA* WT HRD-high tumors, in which high S3 levels appeared to be associated with better PFI (but no difference for OS). Tumor immune profile seems to have a greater influence on the prognosis of *BRCA*-mutated and *BRCA* WT HRD-high tumors (compared to *BRCA* WT HRD-low cases), since high expression of B cell lineage, dendritic cells, cytotoxic T lymphocytes, T cells, and CD8 T cell signatures appear to be more strongly associated with favorable survival in these groups of tumors (Fig. 5C-D). Other cellular components of the tumor microenvironment such as fibroblasts or endothelial cells seem to be associated with particularly poor prognosis in *BRCA* WT HRD-high tumors, compared to other ER + /HER2- eBC. However, it should be noted that the prognostic impact of these biological factors remains of lesser magnitude than that of clinical variables (T and N stage), and molecular intrinsic subtypes according to PAM50, as classically described. Same exploratory analysis of variables associated with PFI and OS is also presented in Additional file 12: supplemental figure 10A–B (S10 A–B), but by adjusting each variable on T and N stage.

## Discussion

In this work, carried out using public data from the TCGA, we show that there is a group of ER + /HER2- tumors without *BRCA* mutation, but which nevertheless has a high HRD score. The immunological, transcriptomic, and mutational landscapes of these tumors appear to be different from *BRCA*-proficient, HRD-low tumors, and actually more closely resemble the profile observed in *BRCA 1/2*-mutated tumors.

PARPi such as olaparib and talazoparib have been shown to be effective, with a gain in PFS compared to standard chemotherapy in patients with metastatic breast cancer associated with germline mutations of *BRCA 1/2* [5, 6]. About 7% of patients with breast cancer have a germline *BRCA 1/2* mutation, including 10–15% of patients with TNBC. For this reason, the clinical development of PARPi in breast cancer has mainly concerned TNBC, or in the metastatic setting, only tumors occurring in a context of *BRCA 1/2* germline mutation, by considering these tumors as part of the same “*BRCA*-mutated” entity.

However, it appears that beyond *BRCA 1/2* mutations, a broader spectrum of tumors (“*BRCA*ness”) also has HRD, including tumors with genomic abnormalities (germinal or somatic) involving other genes participating in homologous repair mechanisms (*RAD51C, PALB2, BARD1, RAD51D, and CHEK2, ATM, BAP1, CDK12, and FANCM*) [22]. In breast cancer, this has been described again mainly in TNBC [23–25].

HRD score is a metric that combines the measure of loss of heterozygosity (LOH), telomeric allelic imbalance (TAI), and large-scale state transition (LST), three measures that reflect chromosomal instability [26, 27]. Each of these 3 parameters is associated with the existence of *BRCA 1/2* mutations, but the combination of the 3 makes it possible to make a broader distinction between tumors with and without HRD. The cutoff value beyond which a tumor is considered to be HRD-high was defined by analyzing HRD scores in a training cohort of 1058 breast and ovarian tumors with known *BRCA 1/2* status, and identifying a cutoff of 42 with 95% sensitivity to detect tumors with *BRCA 1/2* mutations or *BRCA 1* promoter methylation[17]. We therefore considered in our work that a HRD score > 42 in tumors without *BRCA* mutations reflected significantly HR-deficient tumors, regardless of the causal mechanism. However, it is probable that the best cutoff of HRD score will vary according to the type of cancer studied, and the objective (identification of *BRCA*-mutated cases, or cases likely to respond to treatment with a DNA-damaging agent, etc.). Accordingly, several clinical studies have investigated tumor sensitivity to platinum salts as a function of various HRD genomic scores beyond *BRCA 1/2* mutations. In the neoadjuvant setting, the addition of platinum to standard chemotherapy improved the rate of pathological complete response (pCR) in patients with tumors having high HRD score, including WT *BRCA 1/2* cases [17]. In the metastatic context, again, most of the available data relate to TNBC. In the TBRC009 trial [28], HRD scores of responders to platinum monochemotherapy were significantly higher, regardless of *BRCA* mutational status. However, these data were not confirmed in the first-line TNT trial comparing carboplatin *vs* docetaxel [29], where only patients with *gBRCA* mutation had a significantly higher response rate with carboplatin (but not patients with a high HRD score). However, tumor analysis was done on the primary tumor, therefore potentially different biologically from the metastatic disease, which may explain these results. These findings suggest that the HRD assay is promising in concept, but whether it can be used to identify somatic or *gBRCA* wild-type patients who may benefit from PARPi or platinum-based therapy remains to be determined.

The potential immunogenicity of tumors with HRD appears to be greater than average. Numerous studies have demonstrated that tumors with an S3 mutational signature had high expression of certain checkpoint inhibitors of the immune response, such as CTLA-4 or PD-L1 [30, 31]. Thus, recent reports have suggested that mutations in HR pathways may positively influence response to ICB [32–34]. In addition, tumors with *BRCA 1/2* mutations, in particular breast cancers, also seem to have higher expression of PD-L1, inflammatory interferon-γ signatures, and stronger infiltration by immune cells, associated with higher mutational load (TMB) [35]. There is therefore a theoretical rationale for using immunotherapy in these *BRCA*-mutated tumors, or tumors with *BRCA*ness. This is currently being tested in an ongoing breast cancer trial (NCT03025035).

In addition, preclinical work indicates that part of the antitumor effect of PARPi is linked to the activation of immune response, in particular via activation of the STING pathway [36, 37]. The combination of immunotherapy plus PARPi therefore appears attractive and has given encouraging initial clinical results [38], including for patients with *BRCA* WT tumors [39, 40]. Numerous clinical studies are currently testing this concept (NCT02484404, NCT03594396, and NCT03167619), but without prior definition of the population likely to benefit from these associations.

Our study seems to show that the immune context of ER + /HER2- *BRCA* WT breast cancers with a high HRD score closely resembles that of mutated *BRCA* cancers and theoretically would therefore be more favorable to response to checkpoint inhibitors than *BRCA* WT HRD-low tumors. Our results raise the question of the value of immunotherapy, and of PARPi + ICB combinations for this very specific population that we have characterized.

Lastly, when compared to *BRCA*-mutated or *BRCA* WT HRD-low tumors, our study revealed that *BRCA* WT HRD-high breast tumors showed specific genomic and transcriptomic features, including differences in the expression of genes involved in estrogen response, immune response, and proliferation/cell-cycle pathways. These results may help to provide avenues for specifically considering certain targeted therapies in this tumor subtype, such as anti-CDK 4/6, and possibly combining them with immunotherapy approaches. Preclinical studies indeed showed a rationale for combining these treatments with ICB [41, 42], but here again, ongoing phase I/II combination studies (NCT03685331, NCT04481113, NCT01434316) proposed the combination without prior selection of patients based on any biomarker. Likewise, in vitro, CDK 4/6 inhibitor + PARPi combinations have been shown to be synergistic on breast cancer tumor lines [43]. In vitro and in vivo ovarian cancer models combining PARPi with CDK 4/6 showed synergistic effects with palbociclib by inducing HRR deficiency through downregulation of MYC-regulated HR pathway genes, leading to synthetic lethality with olaparib [44]. In clinical trials, a combination of olaparib, palbociclib, and fulvestrant is being investigated in patients with *BRCA*-mutated metastatic breast cancer (NCT03685331).

However, despite the biological differences that we highlight in this work, the prognosis of these ER + /HER2- BRCA-proficient HRD-high tumors does not seem to differ from BRCA-proficient HRD-low tumors. The question of a possible prognostic impact of BRCA mutations and/or HRD parameters in breast cancer remains a matter of debate: Higher HRD scores are associated with a higher probability of pCR after neoadjuvant chemotherapy in early TNBC, but also seem to be associated with better outcome here again in TNBC treated with adjuvant doxorubicin/cyclophosphamide [45]. However, patients with breast cancer who carry a germline BRCA mutation seem to have similar survival as non-carriers [46]. Our results seem to indicate that in ER + /HER2- breast cancer, HRD score is not prognostic by itself. In our study, the outcome of BRCA-proficient HRD-high tumor seems quite good, so the question of additional therapies for these patients could eventually lead to overtreatment. These results of survival should, however, be interpreted with caution, insofar as the TCGA database is poorly informed with regard to the adjuvant treatments received by the patients, and which are important for their long-term outcome. It could be interesting to validate our biological discoveries on an independent series of patients for whom the data of tumor biology, exhaustive adjuvant treatments, and long-term follow-up are available.

In conclusion, while definitive evidence will require prospective clinical evaluation of PARPi and/or ICB and/or CDK 4/6 inhibitor combinations in cohorts of patients with HRD-high and HRD-low *vs BRCA1/2*-mutated ER + breast cancer, our results could have important implications for translational research and/or the design of future clinical trials and highlight possible novel biological differences among ER + /HER2- breast cancer related to their ability to respond to DNA-damaging agents and ICB.

## Materials and methods

### Patient material

RNAseqV2 data with RSEM normalization, MAF, and corresponding clinical data were downloaded from the TCGA data portal (https://portal.gdc.cancer.gov/). Tumor mutational burden (TMB) for each patient was downloaded from the National Cancer Institute [47]. Homologous recombination deficiency (HRD) scores and the 3 components of HRD/genome scarring scores, namely HRD loss of heterozygosity (LOH), large-scale transition (LST), and telomeric allelic imbalance (TAI), were calculated by Knijnenburg et al.[21].

Mutational signatures were generated using DeconstructSigs [48] (v1.8.0) and COSMIC signatures identified by Alexandrov et al. [49]. Somatic and germline pathogenic mutations of *BRCA 1* and *BRCA 2* were obtained from Kraya et al. [50]. PAM50 subtypes were taken from Knijnenburg et *al.*[21]*.* Thirty-seven driver genes were identified using MutSigCV [51]. Genes with q < 0.1 and that were mutated in at least 1% of the TCGA cohort were considered significantly mutated. A 40-gene signature specific to lymphocyte, myeloid, stromal, and cancer cells was also generated [52]. In brief, 10 genes were selected as markers, respectively, of lymphocyte, myeloid, stromal, and cancer cells and their RNA expression was averaged for each sample to compute a score for each cell type. TIL score was then defined as the ratio of the sum of lymphoid and myeloid scores to the total of lymphoid, myeloid, stromal, and cancer scores.

Abundances for 8 major immune and 2 stromal cell populations were estimated for each patient based on RSEM log2-transformed gene expressions and using “MCPcounter” R package [53]. Five metagenes: IFN-$\gamma$, immune checkpoints (ICK), cytotoxicity (CYTOX), Th1 orientation (Th1), and cytotoxic lymphocytes (CTL), were calculated by averaging the RSEM log2-transformed gene expressions of the corresponding component genes [54–56] (Additional file 13: supplemental file 1).

### Statistical analysis

Patient and disease characteristics were compared across the different groups of interest using the chi-square or Fisher’s exact test for qualitative variables and the Wilcoxon test for continuous variables, as appropriate. All correlations were computed using Pearson’s correlation coefficient.

The HRD score and *BRCA 1/2* mutation status were used to categorize patients into three groups: Patients with a *BRCA 1/2* mutation comprised the mutated group, patients wild type for *BRCA 1/2* with a low (≤ 42) (or high > 42) HRD score were, respectively, classed in the *WT* HRD-low (*WT* HRD-high) group (Additional file 14: supplemental file 2).

Univariate and multivariate Cox regression models were used to estimate hazard ratios (HR) and 95% confidence intervals (CIs) for overall survival (OS) and progression-free interval (PFI). Survival curves were estimated by the Kaplan–Meier method and compared with the Log-rank test (univariate analysis). Optimal cutoffs for gene expression were estimated based on maximally selected rank statistics [57]. Multivariate Cox models were fitted including variables with p values < 0.10 in univariate models using lasso penalty. To limit optimism and overfitting, 500 bootstrap samples were generated, and only variables mostly (> 90%) selected through the 500 corresponding lasso Cox models were kept in the final model. Then, mean hazard ratios (HR) with confidence intervals based on 5th and 95th percentiles were estimated over bootstrap samples. Note that survival information was missing for one patient of the ER + /HER2- cohort.

Differential expression analysis was performed with the DESeq2 R package [58]. Three groups were compared. Raw p values associated with each gene were adjusted using Bonferroni correction, as advised. Gene Set Enrichment Analysis (GSEA) was used to identify biological pathways that are enriched in the lists of differential gene characteristics of each group of patients. Pathways from the Hallmark database were used. GSEA was conducted using clusterProfiler R package [59].

Statistical analyses were performed using R software (http://www.R-project.org/), and graphs were drawn using GraphPad Prism version 9.3.1 (GraphPad Software, LLC, San Diego, USA).

## Supplementary Information

**Additional file 1: Supplemental Table 1:** Characteristics of patients under study, whole cohort and ER+/HER2- cohort.

**Additional file 2: Supplemental figure 1 (S1):** A–C. Violin plots representing the distribution of HRD score (A) and signature 3 (C) according to BRCA 1/2 mutational status in the whole cohort (n=928). B–D. Violin plots representing the distribution of HRD score (B) and signature 3 (D) according to PAM50 subtypes and Estrogen Receptor (ER) status considering the whole cohort (n=928). *: Wilcoxon p-value < 0.05.

**Additional file 3: Supplemental figure 2 (S2):** Distributions of HRD score and S3 proportion according to BRCA 1/2 mutational status considering the whole cohort (n = 928) or by breast cancer subtypes (standard pathological classification or PAM50 subtypes). A–B. Violin plots representing the distribution of HRD score (A) and signature 3 proportion (B) according to breast cancer standard pathological classification and BRCA 1/2 mutational status. *: Wilcoxon p-value < 0.05. C–D. Violin plots representing the distribution of HRD score (C) and signature 3 proportion (D) according to PAM50 subtypes and BRCA 1/2 mutational status. *: Wilcoxon p-value < 0.05. E–F. Violin plots representing the distribution of HRD (E) and signature 3 proportion (F) score according to BRCA 1/2 mutational status.

**Additional file 4: Supplemental figure 3 (S3):** A. Violin plots representing the distribution of HRD score according to PAM50 subtypes and BRCA 1/2 mutational status in ER+/HER2- tumors (n = 606). *: Wilcoxon p-value < 0.05. B. Violin plots representing the distribution of signature 3 proportion according to PAM50 subtypes and BRCA 1/2 mutational status in ER+/HER2- tumors (n=606). *: Wilcoxon p-value < 0.05.

**Additional file 5: Supplemental figure 4 (S4):** A. Donuts representing the repartition of HRD score, PAM50 subtype, BRCA 1/2 mutations, Signature 3 proportion and ER status in the whole cohort (n=928). B–D. Diagrams representing the proportion of the different PAM50 subtypes among the BRCA 1/2 mutated (B), BRCA 1/2 WT HRD-high (C) and BRCA 1/2 WT HRD-low (D) populations in ER+/HER2- tumors (n = 606). *: Wilcoxon p-value < 0.05.

**Additional file 6: Supplemental figure 5 (S5):** Association between HRD score and proliferation signature. A. Dot plot representing HRD score (Y-axis) given proliferation score (X-axis). B. Boxplots representing the distribution of proliferation score according to HRD status. *: Wilcoxon p-value < 0.05. C. Boxplots representing the distribution of proliferation score according to PAM50 and HRD status. *: Wilcoxon p-value < 0.05.

**Additional file 7: Supplemental figure 6 (S6):** Association of genomic features quantifying tumor HRD and immunological characterization in ER+/HER2- tumors (n = 606). Violin plots representing the distribution of HRD, TAI, LST and LOH scores (A), signature 3 proportion (B), and immune signatures (C) according to HRD level and BRCA 1/2 mutational status in ER+/HER2- tumors. *: Wilcoxon p-value < 0.05.

**Additional file 8: Supplemental figure 7 (S7).** Boxplots representing the distribution of BRCA2 gene expression according to HRD and BRCA 1/2 mutational status in ER+/HER2- tumors (n = 606). *: Wilcoxon p-value < 0.05.

**Additional file 9: Supplemental figure 8 (S8):** Boxplots representing the distribution of BRCA 1 (A, C, E, G) and BRCA 2 (B, D, F, H) gene expression according to HRD and BRCA 1/2 mutational status in ER+/HER2- tumors (n = 606) represented by PAM50 subtype. *: Wilcoxon p-value < 0.05.

**Additional file 10: Supplemental figure 9 (S9):** Violin plots representing the number of pathogenic or likely pathogenic somatic mutations associated with homologous recombination (HR) according to HRD and BRCA 1/2 mutational status in ER+/HER2- tumors (n = 606).

**Additional file 11: Supplemental table 2:** Factors associated with Overall survival by univariate and multivariate analysis using Cox models with lasso penalty.

**Additional file 12: Supplemental figure 10 (S10):** Overall survival and progression-free interval according to BRCA 1/2 mutated status and HRD score level in ER+/HER2- tumors (n = 606). All variables were adjusted on T and N stages. A–B. Forest plots of hazard ratio (HR) for the association of the clinical variables and immune scores with overall survival (A) and progression-free interval (B) according to BRCA 1/2 mutational status and HRD score level. Red lines: patients with BRCA mutated tumors, green lines: patients with BRCA WT HRD-high tumors, blue lines: patients with BRCA WT HRD-low tumors. Horizontal lines represent 95% CI. Each point represents estimated HR. The dashed vertical line indicates HR = 1. *: Wald-test p-value < 0.05.

**Additional file 13: Supplemental file 1:** Presentation of the five meta-genes used: IFN-γ, immune checkpoints (ICK), cytotoxicity (CYTOX), Th1 orientation (Th1) and cytotoxic lymphocytes (CTL).

**Additional file 14: Supplemental file 2:** The HRD score and BRCA 1/2 mutation status were used to categorize patients into three groups: patients with a BRCA 1/2 mutation comprised the mutated group, patients wild-type for BRCA 1/2 with a low (≤ 42) (or high > 42) HRD score were respectively classed in the WT HRD-low (WT HRD-high) group.

## Data Availability

The datasets analyzed during the current study are available from the corresponding author on reasonable request. The dataset supporting the conclusions of this article is available in the TCGA database portal (https://portal.gdc.cancer.gov/).
