# Supplementary material for: Molecular intrinsic subtypes, genomic, and immune landscapes of BRCA-proficient but HRD-high ER-positive/HER2-negative early breast cancers
Source: Breast Cancer Res. 2022 Nov 18;24:80. doi: 10.1186/s13058-022-01572-6 (PMC9675271; doi:10.1186/s13058-022-01572-6)
Supplement: Supplementary file 1 — Additional file 1: Supplemental Table 1: Characteristics of patients under study, whole cohort and ER+/HER2- cohort. [file 13058_2022_1572_MOESM1_ESM.docx]

**Supplemental Table 1**: Characteristics of patients under study, whole cohort and ER+/HER2- cohort.

| **Whole cohort (n=928)** | |
| --- | --- |
| **Pathological subtypes** | |
| ER+/HER2- | 606 (66%) |
| HER2+ | 157 (17%) |
| TNBC | 165 (17%) |
| **Intrinsic molecular subtypes (according to PAM50)** | |
| Luminal A | 492 (53%) |
| Luminal B | 195 (21%) |
| Basal-like | 164 (18%) |
| HER2-enriched | 77 (8%) |
| **ER+/HER2- cohort (n=606)** | |
| Luminal A | 426 (70%) |
| Luminal B | 158 (26%) |
| Basal-like | 17 (3%) |
| HER2-enriched | 5 (1%) |
| HRD High | 55 (9%) |
| S3 High *(65 missing values)* | 55 (9%) |
| *BRCA*-mutated | 24 (4%) |
| *Germline* | 13 (54%) |
| *Somatic* | 11 (46%) |
| *BRCA* WT/HRD High | 44 (7%) |
| *BRCA* WT/ HRD Low | 538 (89%) |
| *BRCA* WT/ S3 High | 45 (7%) |
| *BRCA* WT/ S3 Low | 472 (78%) |

*TNBC : Triple Negative Breast Cancer, S3 : signature 3, WT : Wild Type*
