# Supplementary figures and images for: Molecular intrinsic subtypes, genomic, and immune landscapes of BRCA-proficient but HRD-high ER-positive/HER2-negative early breast cancers
Source: Breast Cancer Res. 2022 Nov 18;24:80. doi: 10.1186/s13058-022-01572-6 (PMC9675271; doi:10.1186/s13058-022-01572-6)

**A**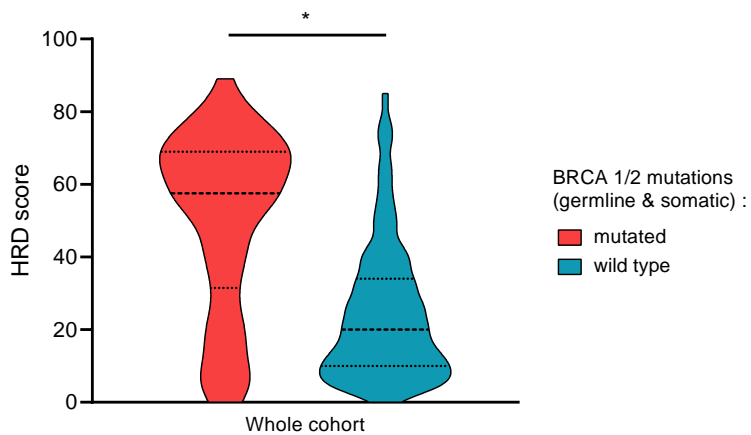**B**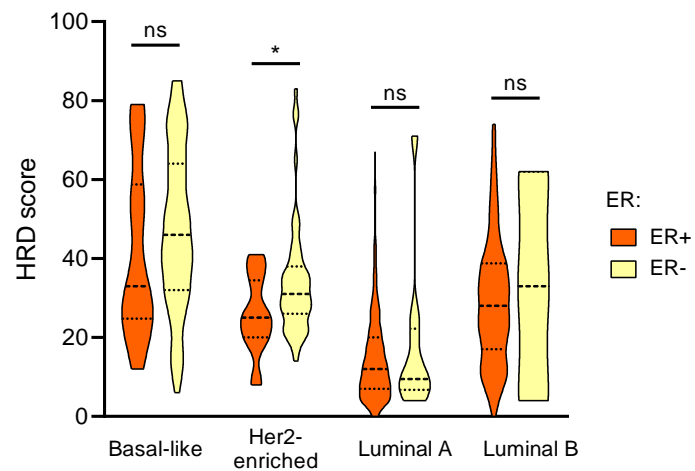**C**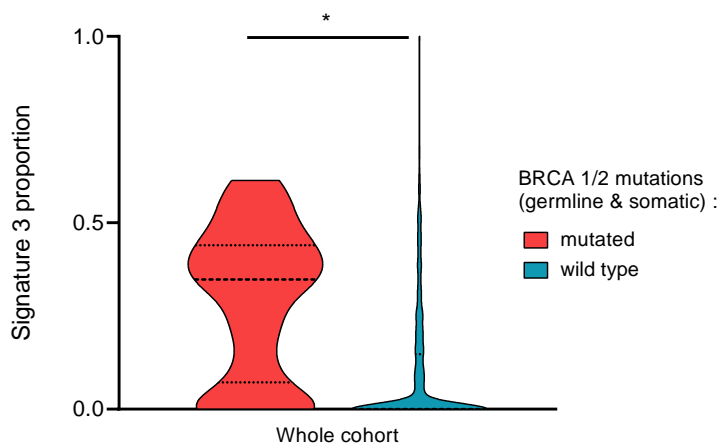**D**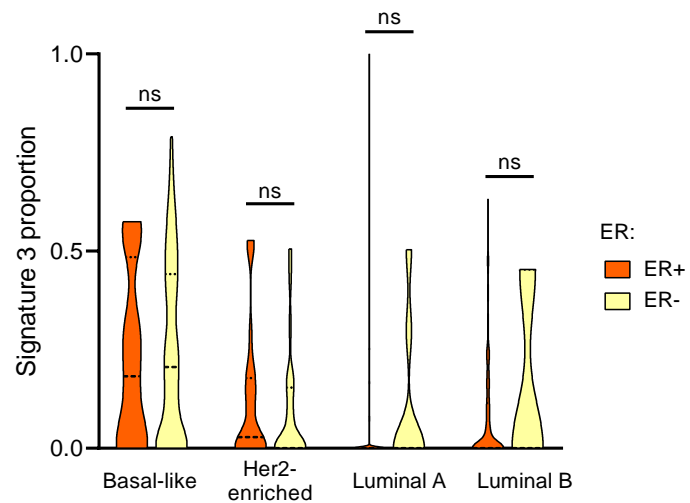

Supplement: Supplementary file 2 — Additional file 2: Supplemental figure 1 (S1): A–C. Violin plots representing the distribution of HRD score (A) and signature 3 (C) according to BRCA 1/2 mutational status in the whole cohort (n=928). B–D. Violin plots representing the distribution of HRD score (B) and signature 3 (D) according to PAM50 subtypes and Estrogen Receptor (ER) status considering the whole cohort (n=928). *: Wilcoxon p-value < 0.05. [file 13058_2022_1572_MOESM2_ESM.pdf]

**A**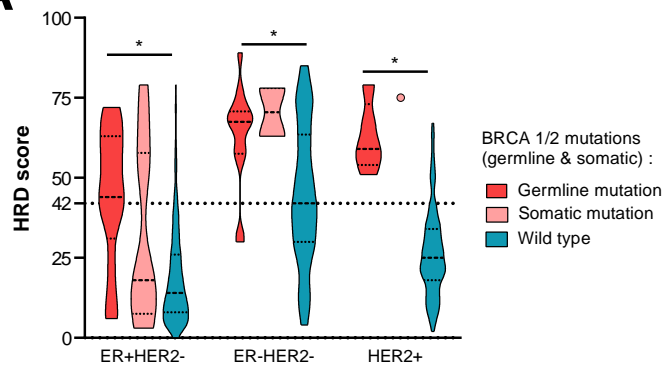**B**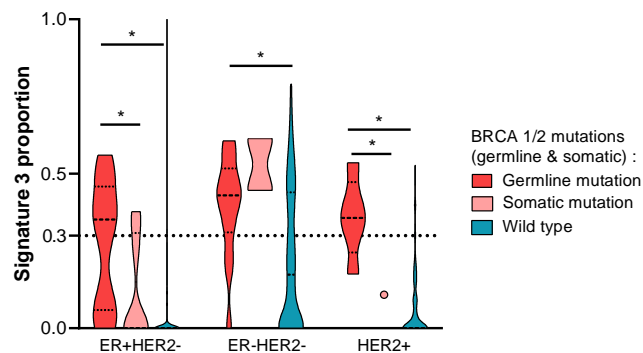**C**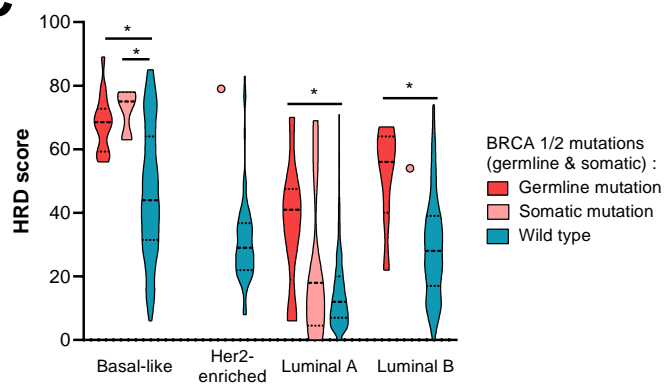**D**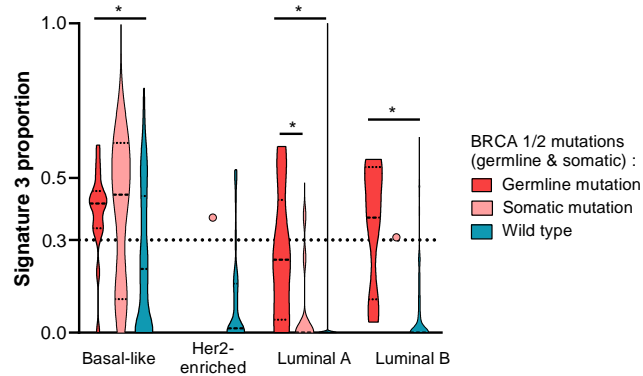**E**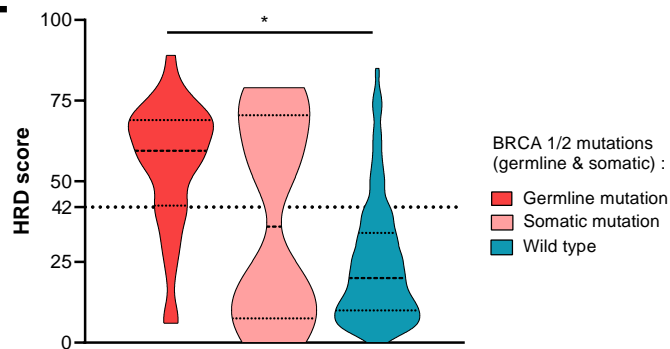**F**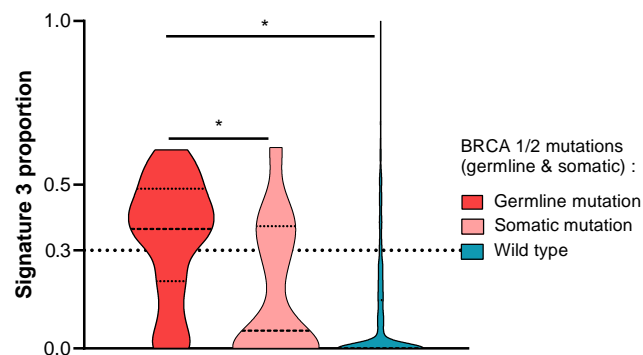

Supplement: Supplementary file 3 — Additional file 3: Supplemental figure 2 (S2): Distributions of HRD score and S3 proportion according to BRCA 1/2 mutational status considering the whole cohort (n = 928) or by breast cancer subtypes (standard pathological classification or PAM50 subtypes). A–B. Violin plots representing the distribution of HRD score (A) and signature 3 proportion (B) according to breast cancer standard pathological classification and BRCA 1/2 mutational status. *: Wilcoxon p-value < 0.05. C–D. Violin plots representing the distribution of HRD score (C) and signature 3 proportion (D) according to PAM50 subtypes and BRCA 1/2 mutational status. *: Wilcoxon p-value < 0.05. E–F. Violin plots representing the distribution of HRD (E) and signature 3 proportion (F) score according to BRCA 1/2 mutational status. [file 13058_2022_1572_MOESM3_ESM.pdf]

**A**

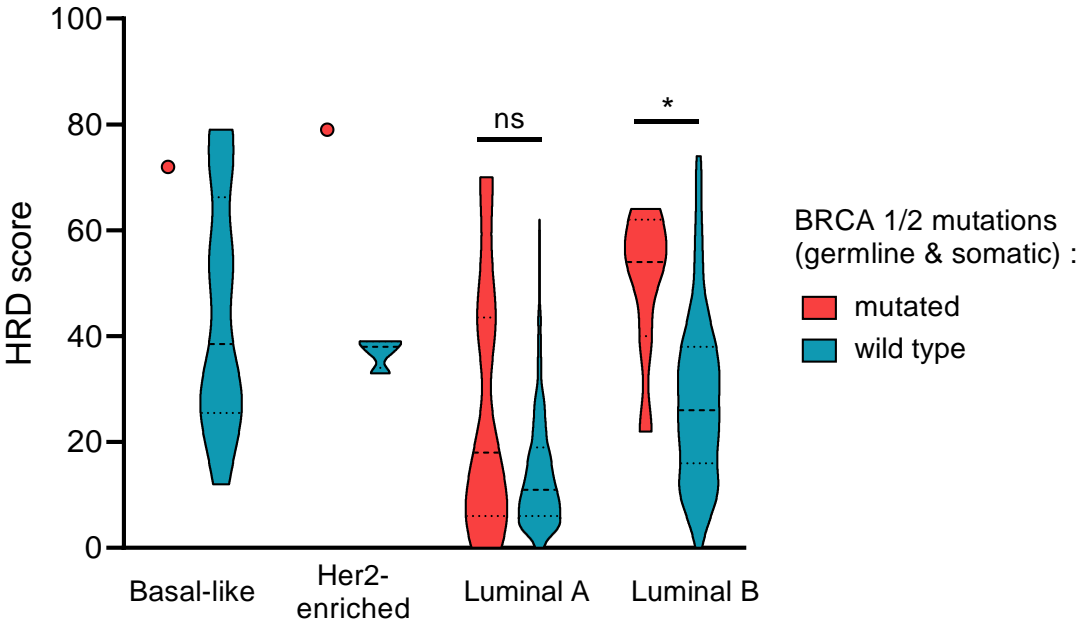

**B**

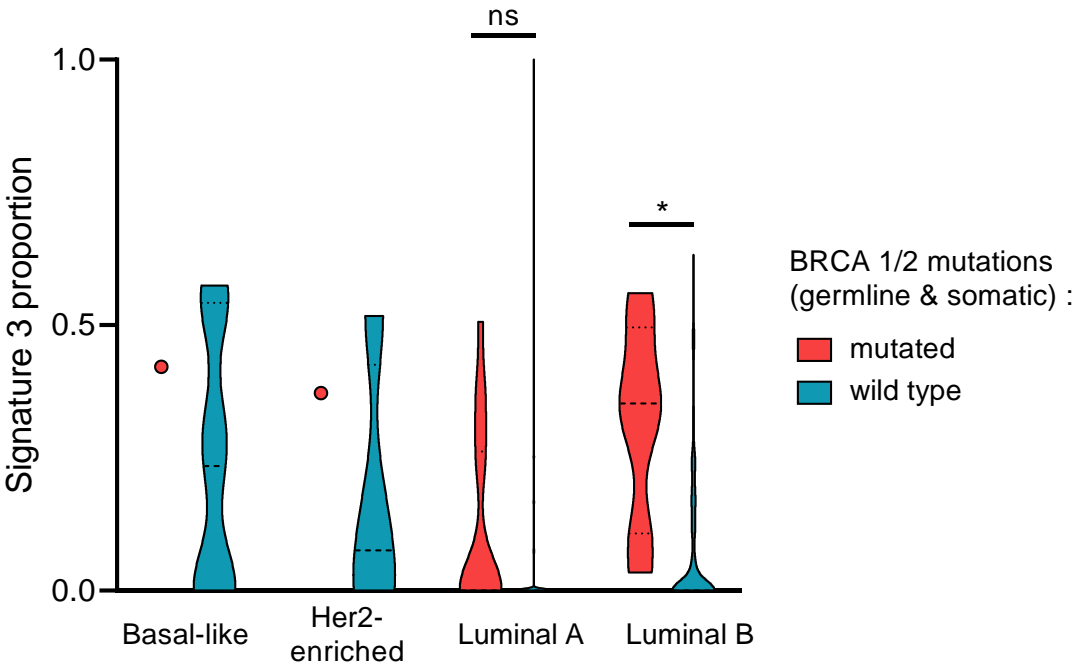

Supplement: Supplementary file 4 — Additional file 4: Supplemental figure 3 (S3): A. Violin plots representing the distribution of HRD score according to PAM50 subtypes and BRCA 1/2 mutational status in ER+/HER2- tumors (n = 606). *: Wilcoxon p-value < 0.05. B. Violin plots representing the distribution of signature 3 proportion according to PAM50 subtypes and BRCA 1/2 mutational status in ER+/HER2- tumors (n=606). *: Wilcoxon p-value < 0.05. [file 13058_2022_1572_MOESM4_ESM.pdf]

**A**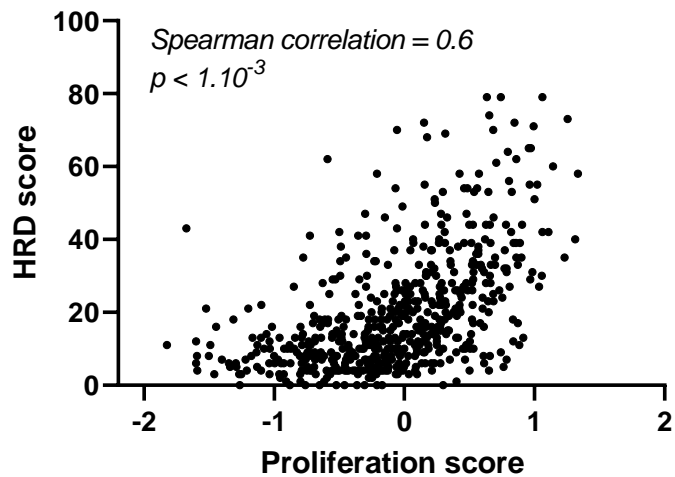**B**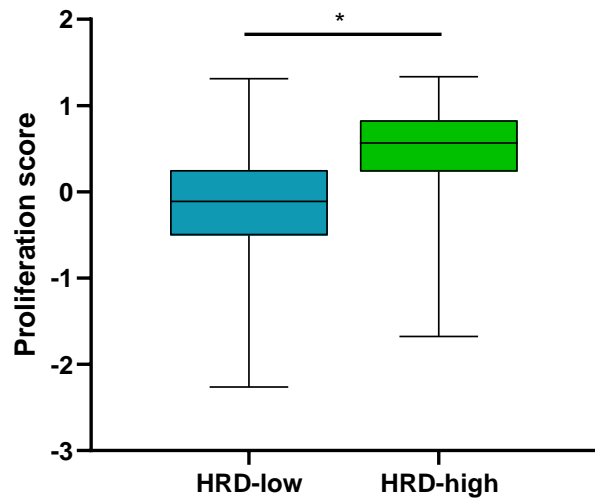**C**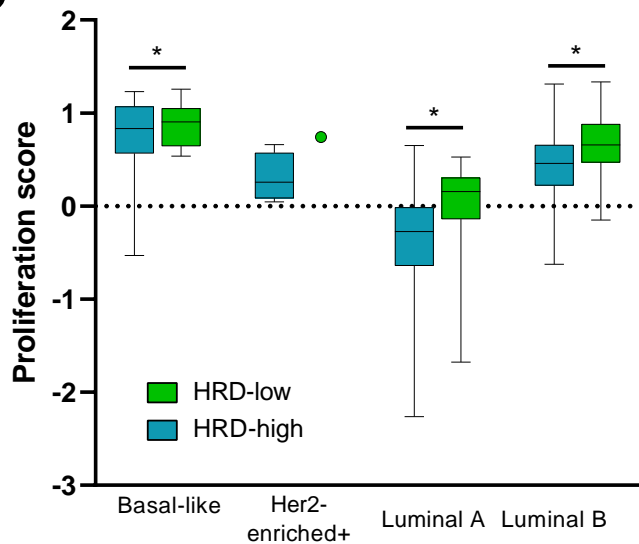

Supplement: Supplementary file 6 — Additional file 6: Supplemental figure 5 (S5): Association between HRD score and proliferation signature. A. Dot plot representing HRD score (Y-axis) given proliferation score (X-axis). B. Boxplots representing the distribution of proliferation score according to HRD status. *: Wilcoxon p-value < 0.05. C. Boxplots representing the distribution of proliferation score according to PAM50 and HRD status. *: Wilcoxon p-value < 0.05. [file 13058_2022_1572_MOESM6_ESM.pdf]

**A**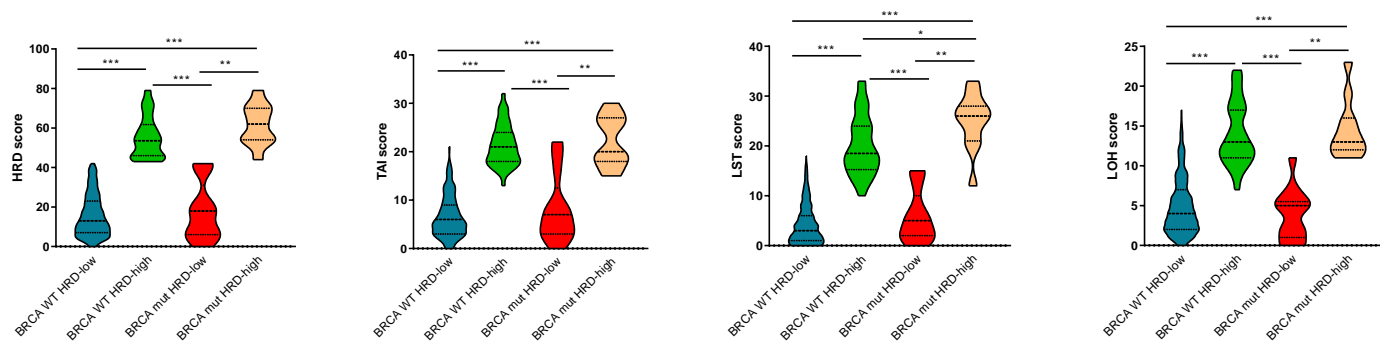**B**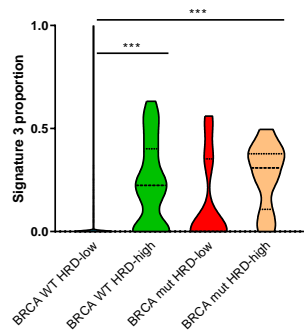**C**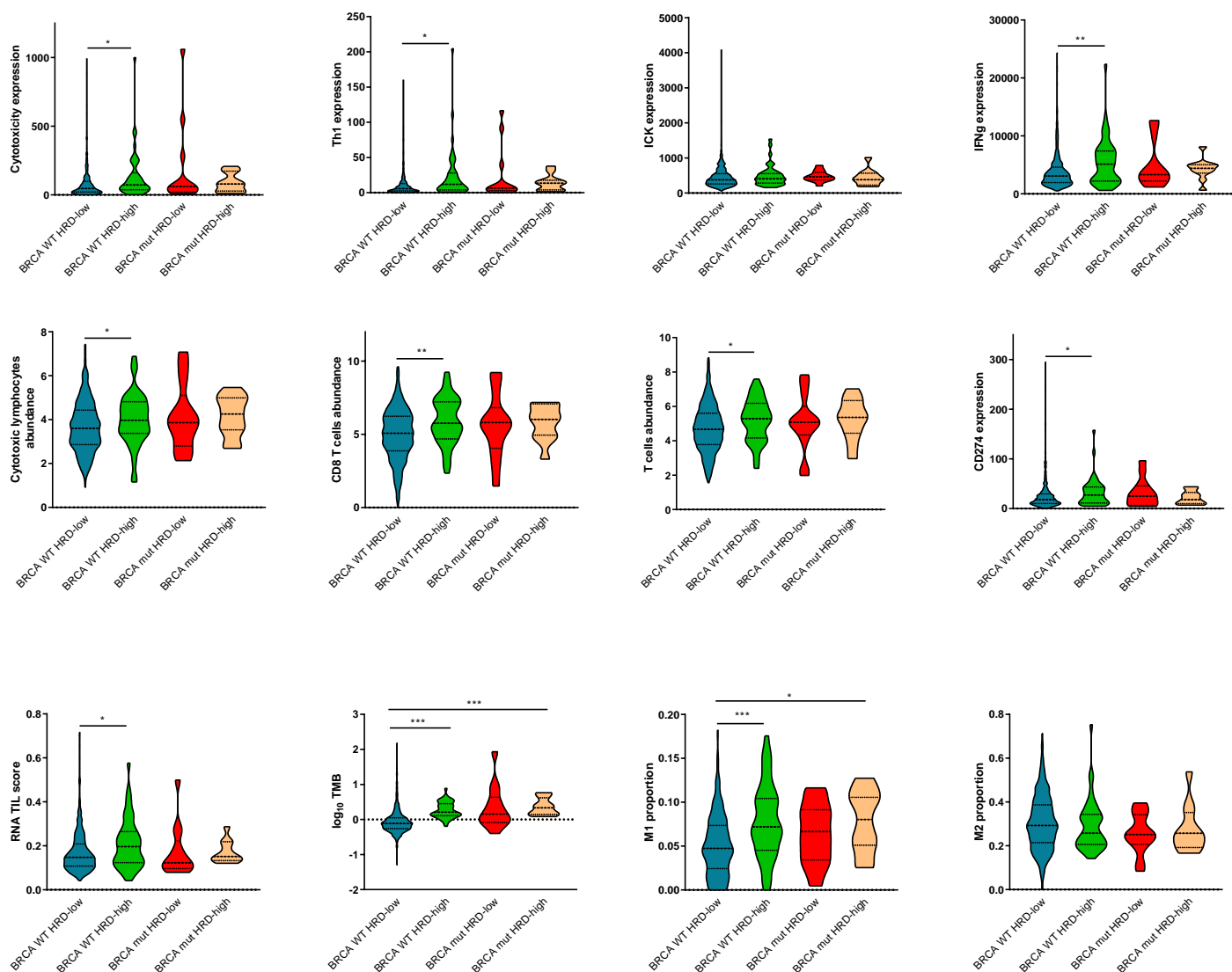

Supplement: Supplementary file 7 — Additional file 7: Supplemental figure 6 (S6): Association of genomic features quantifying tumor HRD and immunological characterization in ER+/HER2- tumors (n = 606). Violin plots representing the distribution of HRD, TAI, LST and LOH scores (A), signature 3 proportion (B), and immune signatures (C) according to HRD level and BRCA 1/2 mutational status in ER+/HER2- tumors. *: Wilcoxon p-value < 0.05. [file 13058_2022_1572_MOESM7_ESM.pdf]

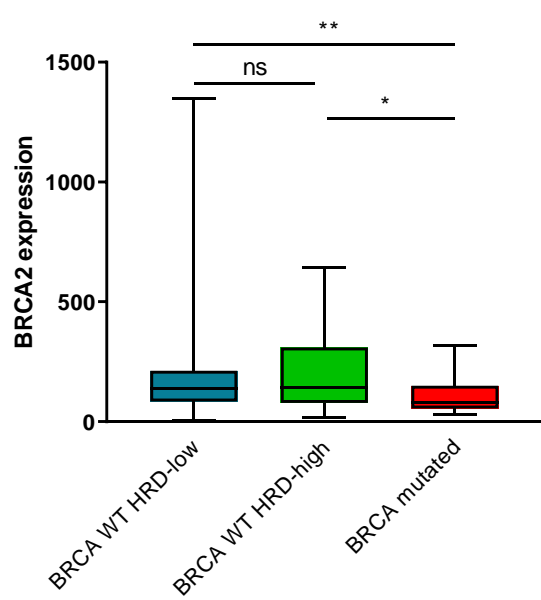

Supplement: Supplementary file 8 — Additional file 8: Supplemental figure 7 (S7). Boxplots representing the distribution of BRCA2 gene expression according to HRD and BRCA 1/2 mutational status in ER+/HER2- tumors (n = 606). *: Wilcoxon p-value < 0.05. [file 13058_2022_1572_MOESM8_ESM.pdf]

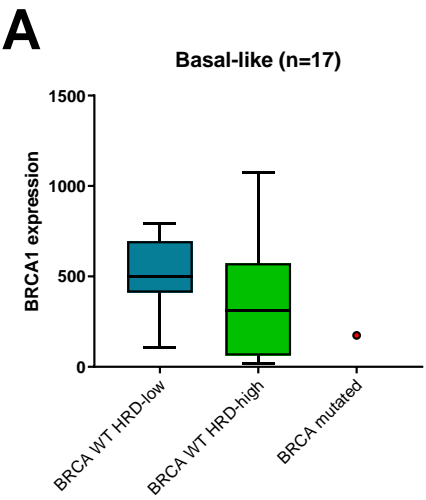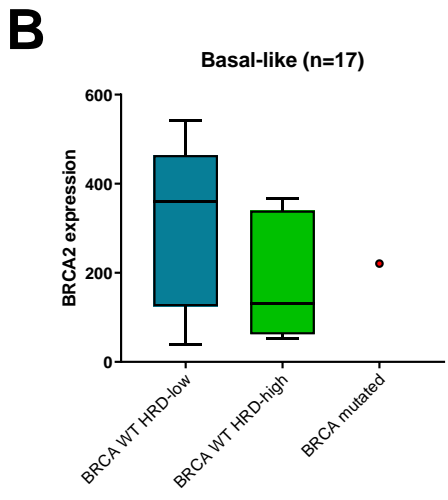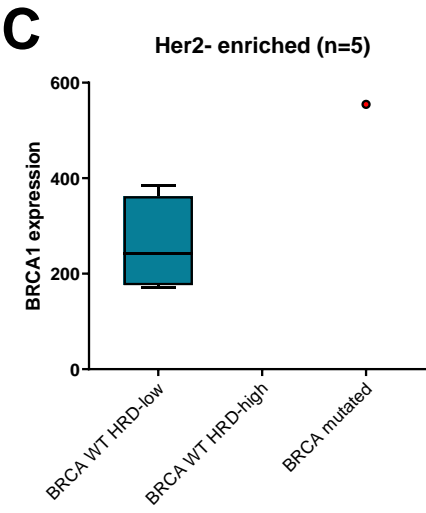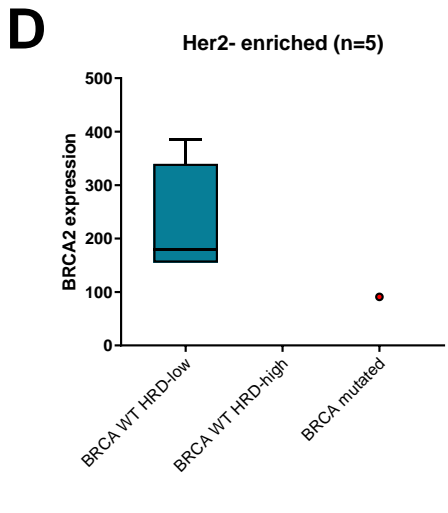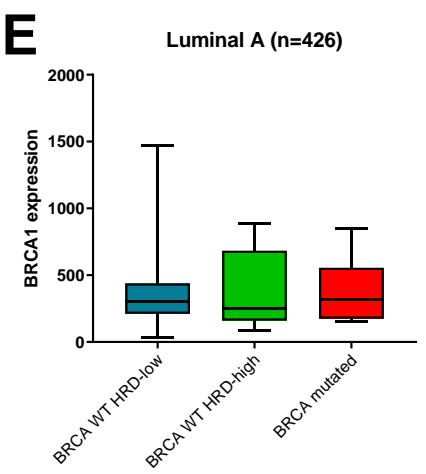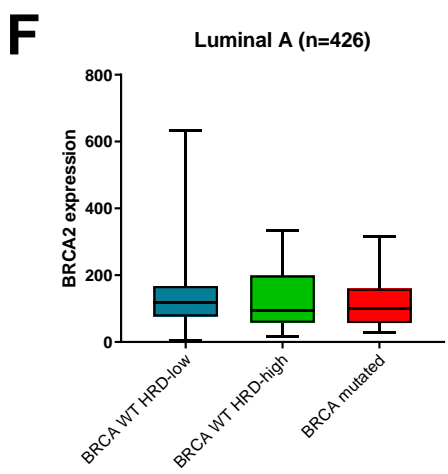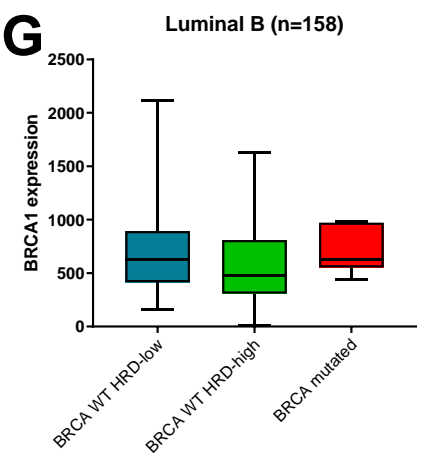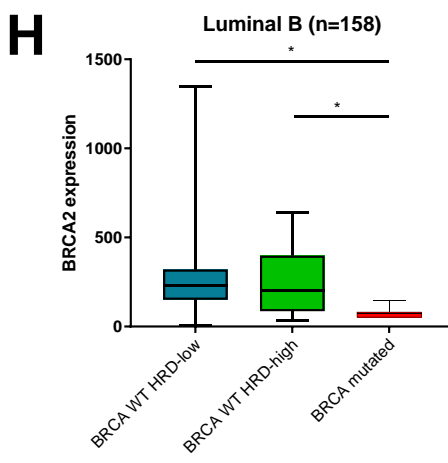

Supplement: Supplementary file 9 — Additional file 9: Supplemental figure 8 (S8): Boxplots representing the distribution of BRCA 1 (A, C, E, G) and BRCA 2 (B, D, F, H) gene expression according to HRD and BRCA 1/2 mutational status in ER+/HER2- tumors (n = 606) represented by PAM50 subtype. *: Wilcoxon p-value < 0.05. [file 13058_2022_1572_MOESM9_ESM.pdf]

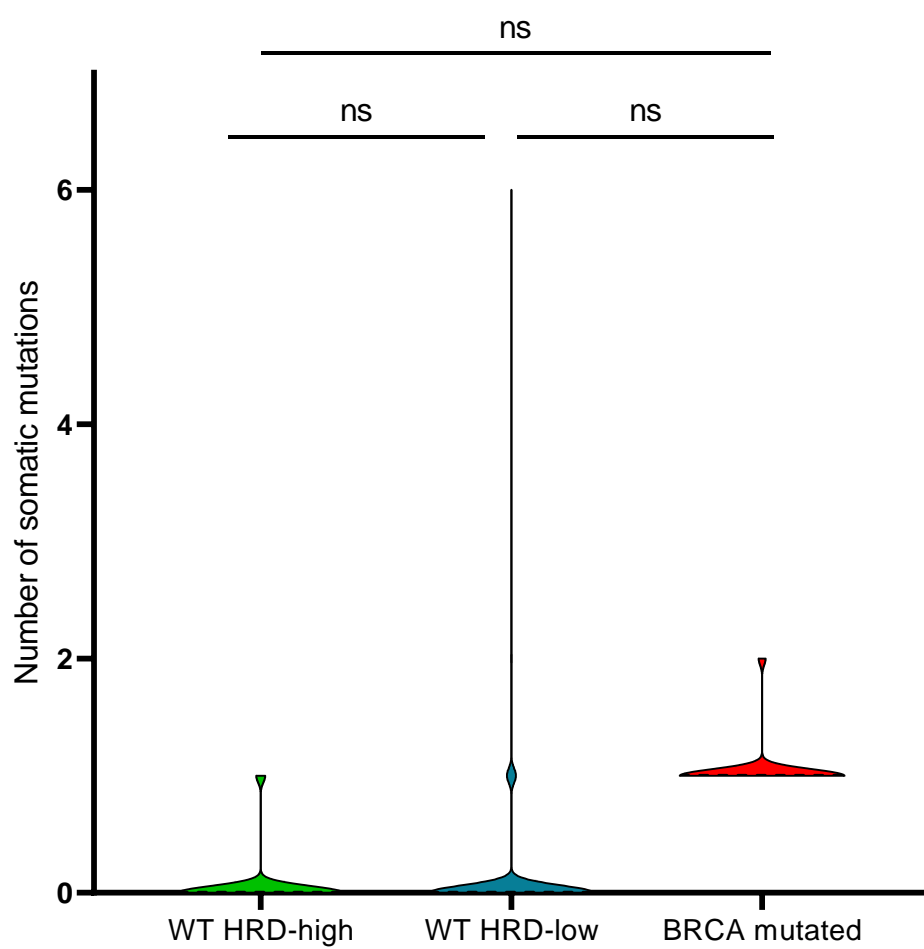

Supplement: Supplementary file 10 — Additional file 10: Supplemental figure 9 (S9): Violin plots representing the number of pathogenic or likely pathogenic somatic mutations associated with homologous recombination (HR) according to HRD and BRCA 1/2 mutational status in ER+/HER2- tumors (n = 606). [file 13058_2022_1572_MOESM10_ESM.pdf]

A

Overall survival - adjusted model

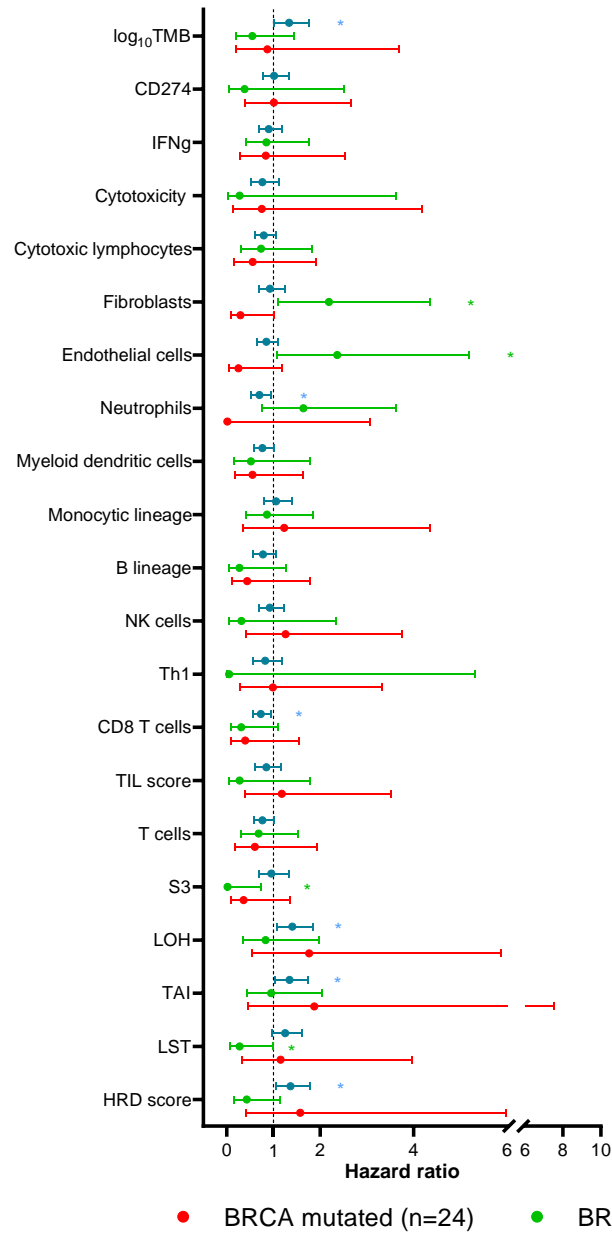

B

Progression-free interval - adjusted model

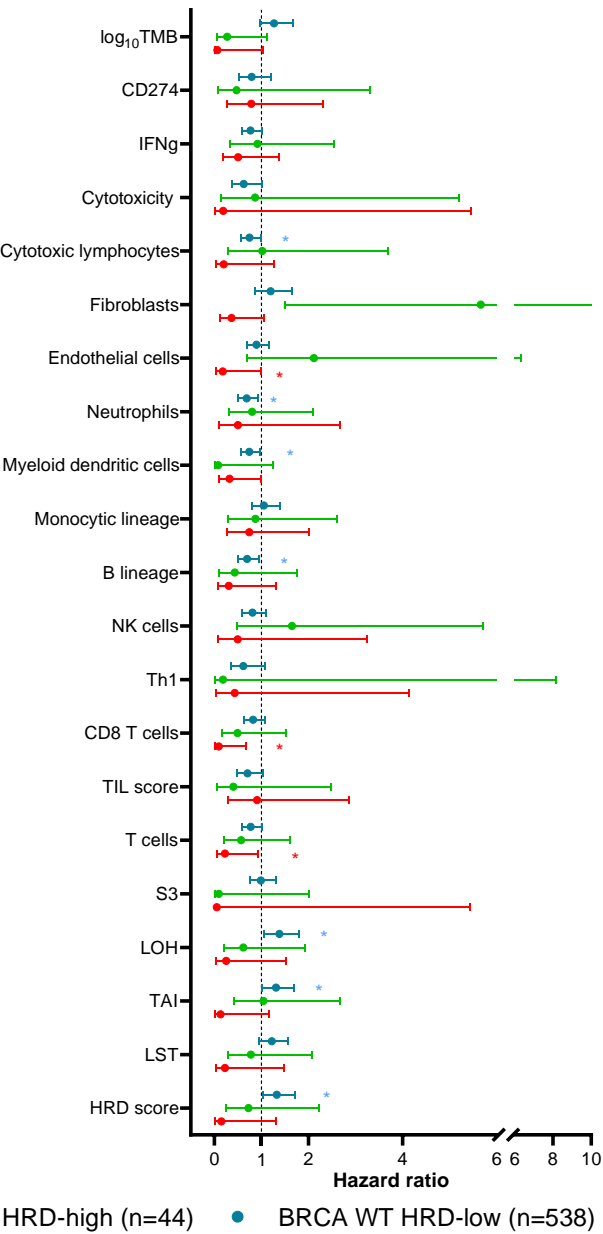

Supplement: Supplementary file 12 — Additional file 12: Supplemental figure 10 (S10): Overall survival and progression-free interval according to BRCA 1/2 mutated status and HRD score level in ER+/HER2- tumors (n = 606). All variables were adjusted on T and N stages. A–B. Forest plots of hazard ratio (HR) for the association of the clinical variables and immune scores with overall survival (A) and progression-free interval (B) according to BRCA 1/2 mutational status and HRD score level. Red lines: patients with BRCA mutated tumors, green lines: patients with BRCA WT HRD-high tumors, blue lines: patients with BRCA WT HRD-low tumors. Horizontal lines represent 95% CI. Each point represents estimated HR. The dashed vertical line indicates HR = 1. *: Wald-test p-value < 0.05. [file 13058_2022_1572_MOESM12_ESM.pdf]
