# Supplementary material for: Molecular intrinsic subtypes, genomic, and immune landscapes of BRCA-proficient but HRD-high ER-positive/HER2-negative early breast cancers
Source: Breast Cancer Res. 2022 Nov 18;24:80. doi: 10.1186/s13058-022-01572-6 (PMC9675271; doi:10.1186/s13058-022-01572-6)
Supplement: Supplementary file 5 — Additional file 5: Supplemental figure 4 (S4): A. Donuts representing the repartition of HRD score, PAM50 subtype, BRCA 1/2 mutations, Signature 3 proportion and ER status in the whole cohort (n=928). B–D. Diagrams representing the proportion of the different PAM50 subtypes among the BRCA 1/2 mutated (B), BRCA 1/2 WT HRD-high (C) and BRCA 1/2 WT HRD-low (D) populations in ER+/HER2- tumors (n = 606). *: Wilcoxon p-value < 0.05. [file 13058_2022_1572_MOESM5_ESM.pdf]

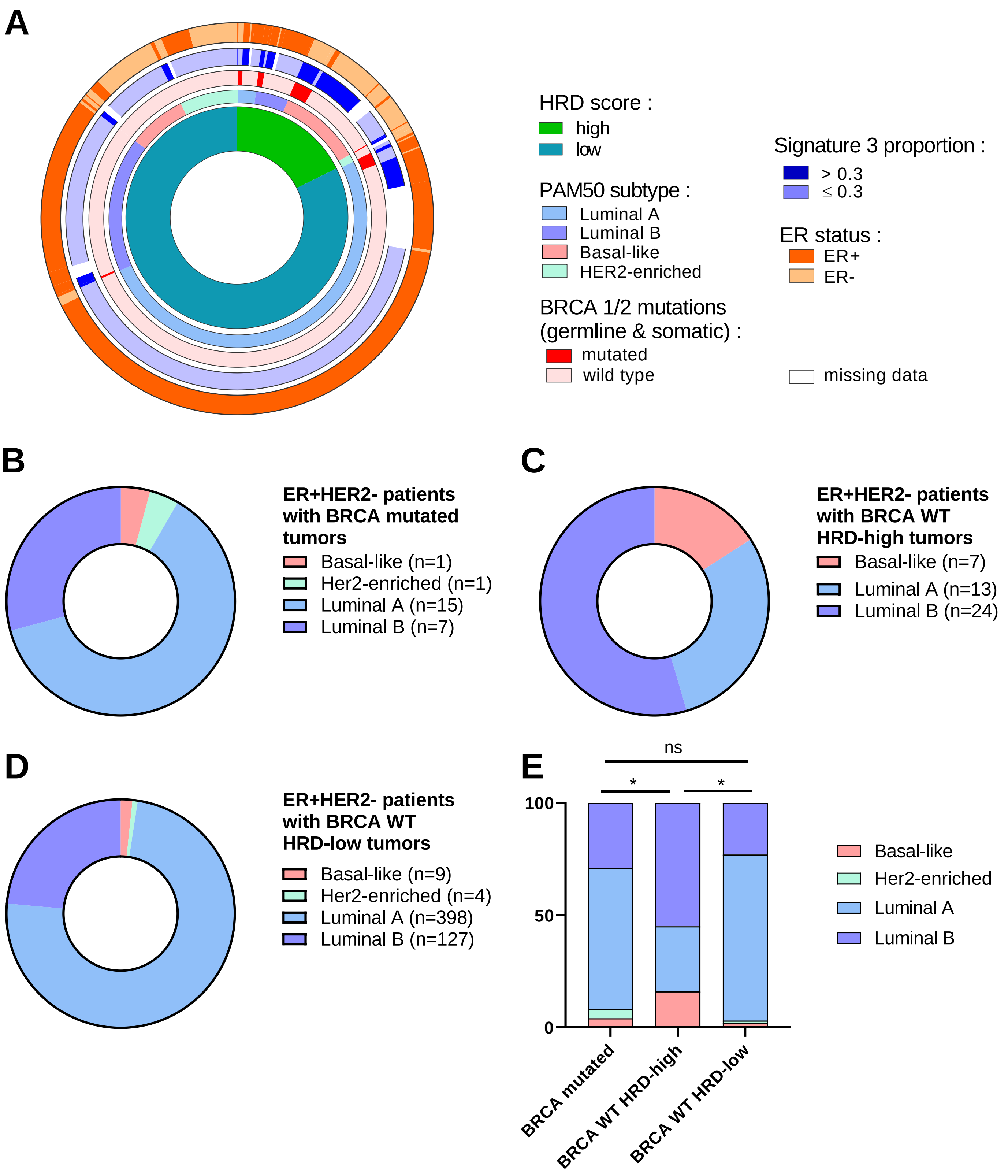
A
HRD score :
high
low
PAM50 subtype :
Luminal A
Luminal B
Basal-like
HER2-enriched
BRCA 1/2 mutations (germline & somatic) :
mutated
wild type
Signature 3 proportion :
> 0.3
≤ 0.3
ER status :
ER+
ER-
missing data
B
ER+HER2- patients with BRCA mutated tumors
Basal-like (n=1)
Her2-enriched (n=1)
Luminal A (n=15)
Luminal B (n=7)
C
ER+HER2- patients with BRCA WT HRD-high tumors
Basal-like (n=7)
Luminal A (n=13)
Luminal B (n=24)
D
ER+HER2- patients with BRCA WT HRD-low tumors
Basal-like (n=9)
Her2-enriched (n=4)
Luminal A (n=398)
Luminal B (n=127)
E
ns
*
*
100
50
0
BRCA mutated
BRCA WT HRD-high
BRCA WT HRD-low
Basal-like
Her2-enriched
Luminal A
Luminal B
