# Supplementary material for: Molecular intrinsic subtypes, genomic, and immune landscapes of BRCA-proficient but HRD-high ER-positive/HER2-negative early breast cancers
Source: Breast Cancer Res. 2022 Nov 18;24:80. doi: 10.1186/s13058-022-01572-6 (PMC9675271; doi:10.1186/s13058-022-01572-6)
Supplement: Supplementary file 11 — Additional file 11: Supplemental table 2: Factors associated with Overall survival by univariate and multivariate analysis using Cox models with lasso penalty. [file 13058_2022_1572_MOESM11_ESM.docx]

**Supplemental Table 2:** Factors associated with Overall survival by univariate and multivariate analysis using Cox models with lasso penalty.

|  |  | Univariate | | | Multivariate |
| --- | --- | --- | --- | --- | --- |
| Variables | Threshold | HR [95 % CI] | p-value | adjusted p-value | HR [95 % CI] |
| Group |  |  |  |  |  |
| *BRCA WT HRD-low* | - | 1 |  |  |  |
| *BRCA WT HRD-high* | - | 1.05 [0.46;2.44] | 0.20 | 0.25 |  |
| *BRCA mutated* | - | 1.94 [0.70;5.34] | 0.90 | 0.9 |  |
| HRD Score | 42 | 1.3 [0.65;2.63] | 0.46 | 0.47 |  |
| TAI Score | 4 | 1.62 [1.02;2.58] | 0.04 | 0.10 | 1.11 [1.10;1.13] |
| LST Score | 9 | 1.61 [1.01;2.58] | 0.05 | 0.10 |  |
| LOH Score | 5 | 1.59 [1;2.51] | 0.05 | 0.10 | 1.17 [1.16;1.19] |
| Signature 3 proportion | 0.3 | 0.7 [0.28;1.74] | 0.44 | 0.47 |  |
| Cytotoxicity | 41.60 | 0.47 [0.29;0.75] | 0.00 | 0.01 |  |
| Th1 | 5.72 | 0.51 [0.31;0.83] | 0.01 | 0.02 |  |
| CTL | 126.23 | 0.52 [0.33;0.83] | 0.01 | 0.02 |  |
| ICK | 505.50 | 0.71 [0.41;1.22] | 0.21 | 0.26 |  |
| IFNg | 11.50 | 0.61 [0.38;0.98] | 0.04 | 0.10 |  |
| CD274 (PD-L1) | 17.04 | 0.65 [0.41;1.04] | 0.07 | 0.11 |  |
| log10 TMB | -0.119186407719209 | 2.03 [1.22;3.38] | 0.01 | 0.02 | 1.66 [1.63;1.70] |
| T cells | 4.20 | 0.39 [0.25;0.63] | 0.00 | 0.00 | 0.46 [0.45;0.46] |
| CD8 T cells | 4.96 | 0.48 [0.3;0.77] | 0.00 | 0.01 | 0.86 [0.85;0.89] |
| Cytotoxic lymphocytes | 3.56 | 0.45 [0.28;0.74] | 0.00 | 0.01 |  |
| NK cells | 1.24 | 0.63 [0.39;1.01] | 0.05 | 0.10 |  |
| B lineage | 2.82 | 0.47 [0.29;0.74] | 0.00 | 0.01 |  |
| Monocytic lineage | 7.83 | 1.2 [0.75;1.92] | 0.44 | 0.47 |  |
| Myeloid dendritic cells | 4.14 | 0.57 [0.35;0.9] | 0.02 | 0.05 |  |
| Neutrophils | 4.90 | 0.53 [0.33;0.85] | 0.01 | 0.03 | 0.57 [0.55;0.58] |
| Endothelial cells | 7.19 | 0.73 [0.45;1.16] | 0.18 | 0.24 |  |
| Fibroblasts | 12.86 | 1.53 [0.96;2.43] | 0.08 | 0.11 | 2.14 [2.05;2.23] |
| TILS | 0.12 | 0.64 [0.4;1.02] | 0.06 | 0.10 |  |

*HR : Hazard Ratio (high vs low level for dichotomized variables), CI: Confidence Interval, adj p-value : adjusted p-value, LOH: Loss Of Heterozygosity, TAI: Telomeric Allelic Imbalance, LST: Large Scale state Transition, CTL : Cytotoxic T cell Lymphocytes, ICK : Inhibitory Immune Checkpoint, IFNg : Interferon gamma, TMB : Tumor Mutational Burden, NK: Natural Killer, TILS : Tumor Infiltrating Lymphocyte signature.*
